# Immune sensing of food allergens promotes avoidance behaviour

**DOI:** 10.1038/s41586-023-06362-4

**Published:** 2023-07-12

**Authors:** Esther B. Florsheim, Nathaniel D. Bachtel, Jaime L. Cullen, Bruna G. C. Lima, Mahdieh Godazgar, Fernando Carvalho, Carolina P. Chatain, Marcelo R. Zimmer, Cuiling Zhang, Gregory Gautier, Pierre Launay, Andrew Wang, Marcelo O. Dietrich, Ruslan Medzhitov

**Affiliations:** 1 Department of Immunobiology, Yale University School of Medicine, New Haven, CT USA; 2 School of Life Sciences, Arizona State University, Tempe, AZ USA; 3 Biodesign Institute, Center for Health Through Microbiomes, Arizona State University, Tempe, AZ USA; 4 Department of Pharmacology, University of São Paulo, São Paulo, Brazil; 5 Department of Comparative Medicine, Yale University School of Medicine, New Haven, CT USA; 6 Centre de Recherche sur l’Inflammation, INSERM UMR1149, CNRS EMR8252, Université Paris Cité, Paris, France; 7 Department of Medicine (Rheumatology, Allergy & Immunology), Yale University School of Medicine, New Haven, CT USA; 8 Howard Hughes Medical Institute, Chevy Chase, MD USA; 9 Tananbaum Center for Theoretical and Analytical Human Biology, Yale University School of Medicine, New Haven, CT USA; 10 Present Address: Department of Immunobiology, Yale University School of Medicine, New Haven, CT USA

**Keywords:** Mucosal immunology, Immunology

## Abstract

In addition to its canonical function of protection from pathogens, the immune system can also alter behaviour^1,2^. The scope and mechanisms of behavioural modifications by the immune system are not yet well understood. Here, using mouse models of food allergy, we show that allergic sensitization drives antigen-specific avoidance behaviour. Allergen ingestion activates brain areas involved in the response to aversive stimuli, including the nucleus of tractus solitarius, parabrachial nucleus and central amygdala. Allergen avoidance requires immunoglobulin E (IgE) antibodies and mast cells but precedes the development of gut allergic inflammation. The ability of allergen-specific IgE and mast cells to promote avoidance requires cysteinyl leukotrienes and growth and differentiation factor 15. Finally, a comparison of C57BL/6 and BALB/c mouse strains revealed a strong effect of the genetic background on the avoidance behaviour. These findings thus point to antigen-specific behavioural modifications that probably evolved to promote niche selection to avoid unfavourable environments.

## Abstract (web summary)

A study using mouse models of food allergy shows that allergic sensitization drives antigen-specific avoidance behaviour mediated by immunoglobulin E antibodies and mast cells.

## Main

Allergies are a class of inflammatory diseases that have increased in prevalence over recent decades^3^. Allergic diseases such as atopic dermatitis, food allergies, asthma and drug hypersensitivities seem to be directly linked to industrialization and urban lifestyles^4^. The physiological roles for these allergic responses, however, remain enigmatic. Type 2 immunity, which includes T helper 2 T cells, IgE antibodies and innate immune cells (for example, mast cells, eosinophils and type 2 innate lymphoid cells), mediates allergic responses. When chronic or excessive, allergic responses become detrimental, and potentially lethal^5^. Allergic responses seem to have an important role in host defence against noxious substances, including venoms, haematophagous fluids, xenobiotics and irritants^6–10^. Indeed, a common feature of allergic responses is the exacerbation of defensive neuronal reflexes such as sneezing, itching and vomiting, which expel harmful substances from the body^11^. In addition to these reflexes, avoidance behaviour was shown to be induced in allergic responses^12–14^, which suggests that type 2 immunity might limit exposure to detrimental stimuli, acting as an efficient defence strategy to prevent further damage. However, the mechanisms by which type 2 responses promote behavioural outputs have yet to be established.

**Fig. 1:**
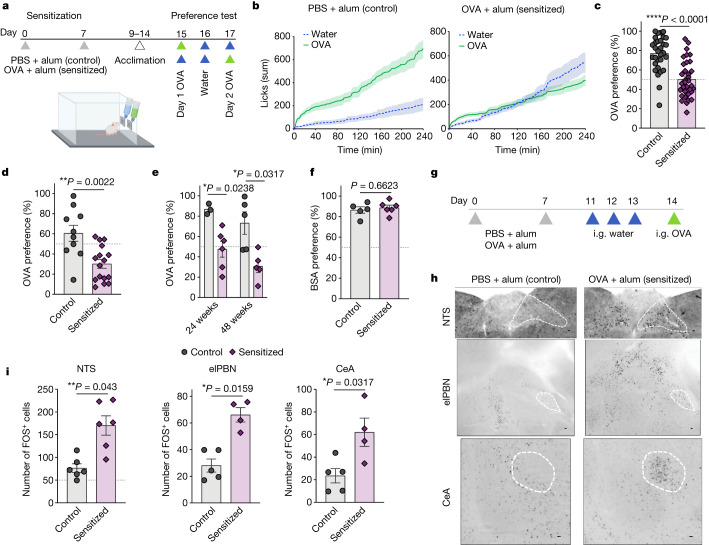
Allergic sensitization induces specific and long-lasting avoidance behaviour to food allergen. **a**, Schematic protocol for allergic sensitization and behavioural assay. **b**, Cumulative licks from mice sensitized with phosphate-buffered saline (PBS) + alum (left) or OVA + alum (right). Preference test consisting of one water bottle and one 1% OVA bottle on day 1 (*n* = 9–10 mice per group). **c**, Preference to OVA solution (*n* = 31 control and 34 allergic mice per group) on day 1 of the test. **d**, Preference to OVA with switched side bottles on day 2 of the test (*n* = 10 control and 16 allergic per group). **e**, Preference to OVA at 24 or 48 weeks after alum or OVA + alum sensitization (*n* = 3–6 mice per group). **f**, Preference to bovine serum albumin (BSA; *n* = 5 control and 6 allergic mice per group). **g**, Schematic protocol of allergic sensitization and oral challenge. Mice were administered intragastric (i.g.) OVA after OVA + alum sensitization and three sham gavages with water. Controls were sensitized with alum alone. **h**, Immunofluorescence images of the NTS (top), elPBN (middle) and CeA (bottom) from control (*n* = 5) or OVA + alum-sensitized (*n* = 4) mice using anti-FOS antibody, 90 min after OVA challenge. Scale bars, 100 µm. **i**, Number of FOS^+^ neurons in the NTS (left), elPBN (middle) and CeA (right) of control or OVA + alum-sensitized mice. Graphs show mean ± s.e.m. *P ≤ 0.05, **P ≤ 0.01, ****P ≤ 0.0001. Two-tailed Mann–Whitney test. Each panel is representative of at least two independent experiments. **a**,**g**, Created with BioRender.com. Source Data

**Extended Data Fig. 1:**
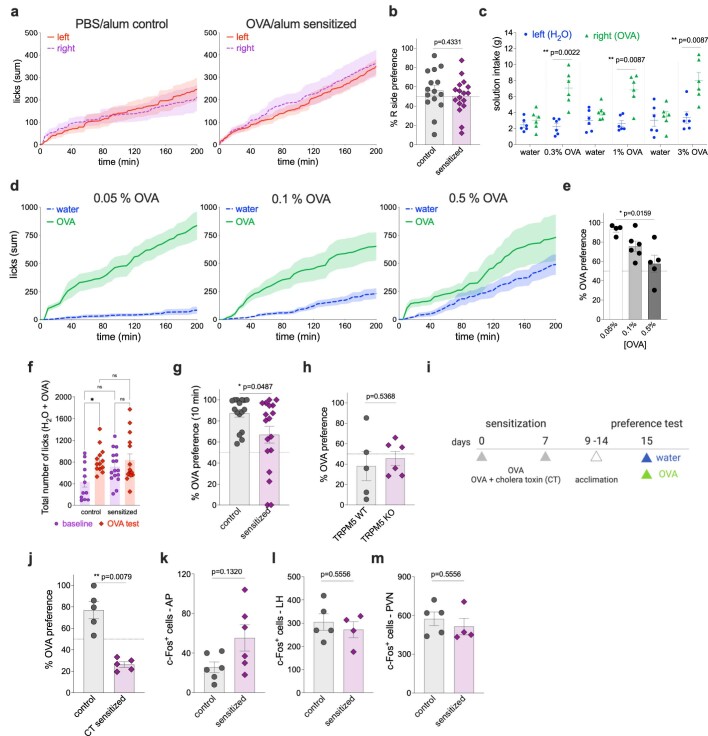
Characterization of immunological avoidance. **a**, Cumulative licks of water solution from right- or left-positioned bottles from control (left) and OVA/alum sensitized BALB/c mice (right) (n = 15-18). **b**, Preference to the water bottle located on the right side by control and OVA/alum sensitized mice (n = 15 control and 17 allergic). **c**, Solution intake of water and varying concentrations of OVA in control mice on day 1 of the two-bottle preference test (n = 6 per concentration). **d**, Cumulative licks to different concentrations of OVA from mice sensitized with OVA/alum (n = 5-6 per concentration). **e**, Preference to different concentrations of OVA in sensitized mice (n = 4-6 per concentration). **f**, Total number of licks during water baseline or OVA test day 1, determined as the sum of licks from the two bottles at 200 min (n = 12-13 control and 15-16 allergic). **g**, Preference to 1% OVA solution within 10 min of offering the two bottles (n = 16 control and 18 allergic). **h**, OVA preference by OVA/alum sensitized TRPM5 WT or KO mice (n = 5 WT and 6 TRPM5 KO). **i**, Experimental protocol for oral sensitization to OVA with cholera toxin (CT). Mice were administered with i.g. OVA with or without CT on days 0 and 7. Controls received OVA alone. **j**, Preference to OVA on day 1 of preference test (n = 5 control and 5 CT sensitized). **k-m**, Total number of cFos+ cells in the area postrema (AP), lateral hypothalamus (LH), and paraventricular nucleus of the hypothalamus (PVN) of OVA/alum sensitized mice (n = 6 control and 6 allergic). Graphs show mean ± s.e.m. *p ≤ 0.05, **p ≤ 0.01, ***p ≤ 0.001. **a-e** and **g-m**, two-tailed Mann-Whitney test, **f**, one-way ANOVA with Dunn’s multiple comparisons test. Each panel is representative of at least two independent experiments. **i**, Created with BioRender.com. Source Data

To examine the effect of allergic sensitization on avoidance behaviour, we sensitized mice with subcutaneous injections of ovalbumin (OVA) and the adjuvant aluminium hydroxide (alum) on days 0 and 7 (Fig. 1a). Control mice received alum without OVA. Mice were then acclimatized to home cages equipped with two lickometers (that is, spouts that automatically detect licks) connected to water bottles. During the acclimation period, mice showed no side preference (Extended Data Fig. 1a,b). After acclimation, we randomly switched the content of one of the bottles to an OVA solution and observed that control mice showed an increased preference for the OVA solution compared to water (Fig. 1b and Extended Data Fig. 1c), suggesting that OVA is appetitive for mice. By contrast, sensitized mice decreased preference for the OVA solution in a dose-dependent manner (Fig. 1b,c and Extended Data Fig. 1d,e). The analysis of the total number of licks indicates that controls approximately double their consumption when OVA is offered compared with baseline water whereas sensitized mice maintain the same total number of licks (Extended Data Fig. 1f), suggesting some regulated mechanism to dilute the allergen concentration by increasing water ingestion. The decreased OVA preference, referred to here as avoidance behaviour, by sensitized mice occurred within 10 min after providing the test bottles (Extended Data Fig. 1g) and persisted on the second day of the test despite the switched bottle sides (Fig. 1d). Notably, avoidance of OVA solution persisted for at least 48 weeks after allergic sensitization (Fig. 1e) and it was specific to OVA, as control and sensitized mice showed comparable preference to a solution containing bovine serum albumin (Fig. 1f). We next found that the transient receptor potential cation channel subfamily M member 5 (TRPM5), required for taste transduction in chemosensory cells^15^, was dispensable for the development of avoidance behaviour (Extended Data Fig. 1h). Finally, allergic sensitization to OVA with oral cholera toxin, an adjuvant known to induce strong humoral but not cellular immune responses in allergy models^16^, also promoted avoidance of OVA (Extended Data Fig. 1i,j). These results indicate that parenteral immunization towards a protein can generate specific avoidance to food, which is consistent with previous observations^12,17^ with the exception that we did not add sucrose to the OVA solution to minimize behavioural and metabolic effects.

Aversive response to unpleasant stimuli was previously shown to induce brain activation within the nucleus of tractus solitarius (NTS), external lateral parabrachial nucleus (elPBN) and central amygdala (CeA)^18,19^. To determine the extent to which the ingestion of allergens can activate these brain areas, we orally challenged control and sensitized mice with OVA and 90 min later collected their brains to test for neuronal activation using FOS as a marker (Fig. 1g). We found that one allergen challenge was enough to induce NTS, elPBN and CeA activation in sensitized mice as compared with controls (Fig. 1h,i). There was no difference in FOS staining of the area postrema, the lateral hypothalamus or the paraventricular nucleus of the hypothalamus (Extended Data Fig. 1k–m). Together, these findings demonstrate that sensitization with OVA leads to prototypical neuronal activation in the central nervous system. These brain regions correlate with avoidant behaviour towards the sensitized protein and are probably triggered as a defence for limiting allergen intake.

**Extended Data Fig. 2:**
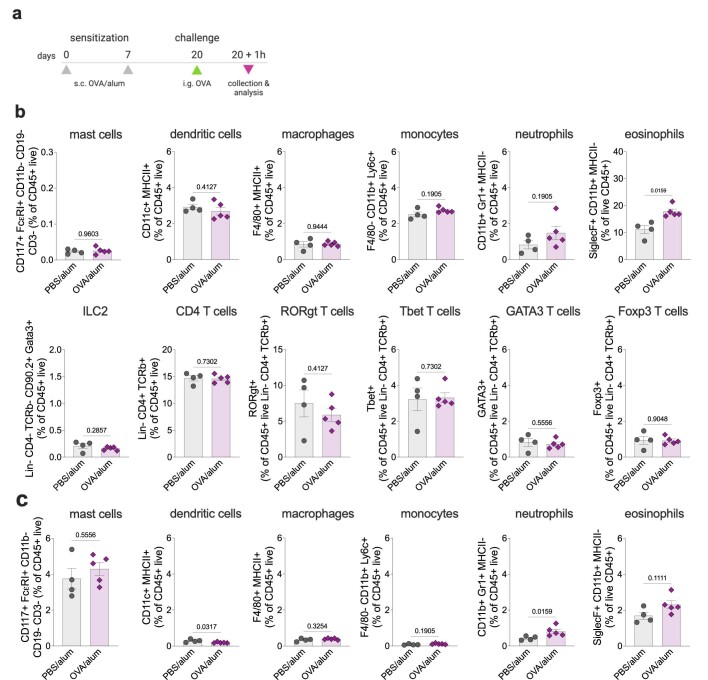
Enteric immune populations. **a**, Experimental protocol to determine cellular inflammation in the small intestines upon oral challenge. BALB/c mice were sensitized with OVA/alum on d0 and d7 and orally challenged with OVA on day 20. One hour later, small intestine cells from the lamina propria **(b)** and epithelial layer **(c)** were isolated and analysed by flow cytometry. Graphs are representative of two independent experiments, n = 4-5 per group. Graphs show mean ± s.e.m. *p ≤ 0.05, **p ≤ 0.01, ***p ≤ 0.001. Two-tailed Mann-Whitney test. Each panel is representative of at least two independent experiments. **a**, Created with BioRender.com. Source Data

**Extended Data Fig. 3:**
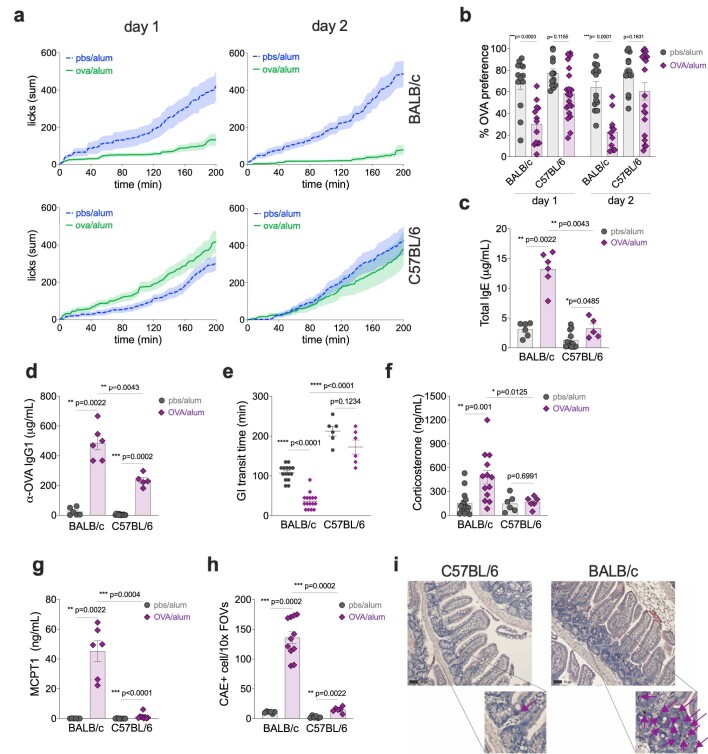
Food avoidance in C57BL/6 strain. **a**, Cumulative licks of OVA solution from BALB/c or C57BL/6 female mice sensitized with PBS/alum or OVA/alum. After day 1, mice received water bottles, and on two-bottle preference test day 2, OVA bottles were offered on the opposite side of d1 (n = 12 control and 8 allergic BALB/cJ, 17 control and allergic C57BL/6J). **b**, Preference to OVA solution (n = 14 control and 13 allergic BALB/cJ, 12 control and 20 allergic C57BL/6J) . **c**, Total serum IgE in mice sensitized with OVA/alum and orally challenged five times (n = 6 control and allergic BALB/cJ, 12 control and 6 allergic C57BL/6J). **d**, OVA-specific IgG1 after allergic sensitization and challenges (n = 6 control and allergic BALB/cJ, 12 control and 5 allergic C57BL/6J). **e**, Gastrointestinal transit time was determined on the fourth oral challenge with OVA in control and OVA/alum sensitized mice using a red carmine assay (n = 15 control and 16 allergic BALB/cJ, 6 control and allergic C57BL/6J). **f**, Systemic corticosterone 1 h after the fifth OVA challenge in control and OVA/alum sensitized BALB/c or C57BL/6 mice (n = 14 control and 13 allergic BALB/cJ, 6 control and allergic C57BL/6J). **g**, MCPT-1 in the serum 1 h after the fifth oral OVA challenge (n = 6 control and allergic BALB/cJ, 12 control and 9 allergic C57BL/6J). **h**, Number of chloroacetate esterase (CAE) positive cells present in the jejunum upon 5 oral challenges of control or allergic mice (n = 6 control and 11 allergic BALB/cJ, 6 control and allergic C57BL/6J). **i**, Representative images of histological sections and CAE staining of OVA/alum-sensitized small intestines (jejunum) from C57BL/6 and BALB/c at magnitude 10x and zoomed images at 40x. Arrows indicate CAE-stained cells. Scale bars = 50 µm. Graphs show mean ± s.e.m. **a** and **c-h**, Two-tailed Mann-Whitney test, **b**, 2-way ANOVA with Turkey’s multiple comparisons test. Each panel is representative of at least two independent experiments. Source Data

Avoidance behaviour towards the sensitized allergen could arise from immune changes in the intestines promoted by skin immunization. Although skin injury was reported to promote expansion of enteric mast cells^20^, we did not find evidence for the accumulation of immune cells in the small intestines after OVA plus alum treatment (Extended Data Fig. 2a–c). We then examined how different genetic backgrounds can affect the development of avoidance behaviour^21,22^. BALB/c and C57BL/6 mice were sensitized with OVA plus alum, and we determined the kinetics of OVA preference. In contrast with sensitized BALB/c mice, C57BL/6 mice did not show strong avoidance behaviour to OVA (Extended Data Fig. 3a,b), as previously suggested^17^. In a food allergy model, C57BL/6 mice mildly increase systemic IgE antibodies compared with allergic BALB/c mice, whereas their allergen-specific IgG1 is similarly induced (Extended Data Fig. 3c,d). As reported previously^22^ and different from BALB/c, allergic C57BL/6 mice do not increase gastrointestinal transit time (Extended Data Fig. 3e) or show signs of diarrhoea (data not shown) following allergen exposure. Finally, allergic sensitized C57BL/6 have no increase in systemic corticosterone levels or mast cell protease 1, and only slight accumulation of mast cells in the small intestine (Extended Data Fig. 3f–i). The comparison between BALB/c and C57BL/6 correlates IgE and mast cells with the development of avoidance behaviour.

**Fig. 2:**
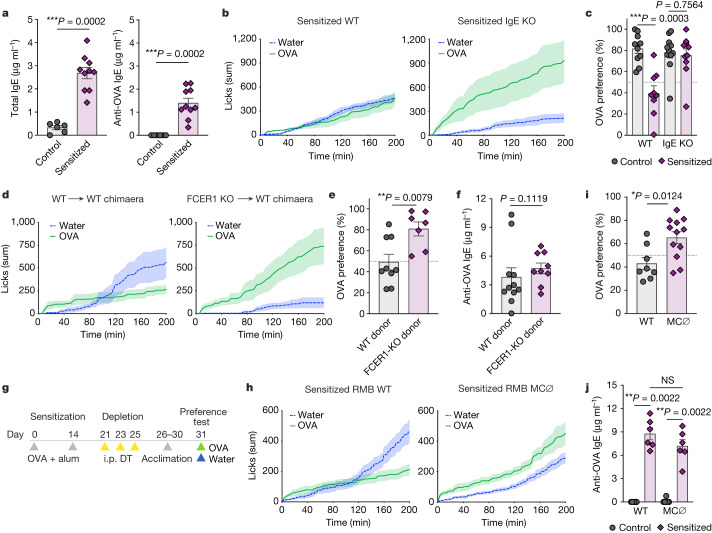
Allergic avoidance requires IgE and mast cells. **a**, Total (left) and OVA-specific (right) levels of serum IgE on day 14 after allergic sensitization in BALB/c mice (*n* = 6 control and 11 allergic mice per group). **b**, Cumulative licks to water and OVA solutions during the two-bottle preference test in OVA + alum-sensitized IgE-KO (right) and wild-type (WT) littermate control (left) mice (*n* = 6 WT and 6 IgE-KO mice per group). **c**, Drinking preference to OVA bottles in controls and allergic sensitized WT or IgE-KO mice (*n* = 9–11 mice per group). **d**, Cumulative licks to water and OVA bottles in OVA + alum-sensitized WT (left) or FCER1 (right) chimaeras. WT or FCER1-KO bone marrow haematopoietic cells were transplanted into irradiated WT recipients (*n* = 5 WT to FCER1 and 6 FCER1 into WT). **e**, Drinking preference to OVA bottles in allergic sensitized WT or FCER1 chimaeras (*n* = 9 WT to FCER1 and 7 FCER1 into WT). **f**, OVA-specific IgE (*n* = 11 WT to FCER1 and 9 FCER1 into WT). **g**, Schematic protocol of FCER1^+^ cell depletion with diphtheria toxin (DT) in RMB BALB/c mice. **h**, Cumulative licks to water and OVA bottles in allergic sensitized and diphtheria toxin-injected RMB WT (left) and RMB mutants (right) (*n* = 9 RMB WT and 14 RMB heterozygotes or mutants). MC ∅, mast cell depleted. **i**,**j**, Preference to OVA solution (*n* = 8 RMB WT and 12 RMB heterozygotes or mutants) (**i**) and OVA-specific IgE (*n* = 6 per group) (**j**) in diphtheria toxin-injected RMB WT and mutants. Graphs show mean ± s.e.m. *P ≤ 0.05, **P ≤ 0.01, ***P ≤ 0.001; NS, not significant. Two-tailed Mann–Whitney test. Each panel is representative of at least two independent experiments. **g**, Created with BioRender.com. Source Data

**Extended Data Fig. 4:**
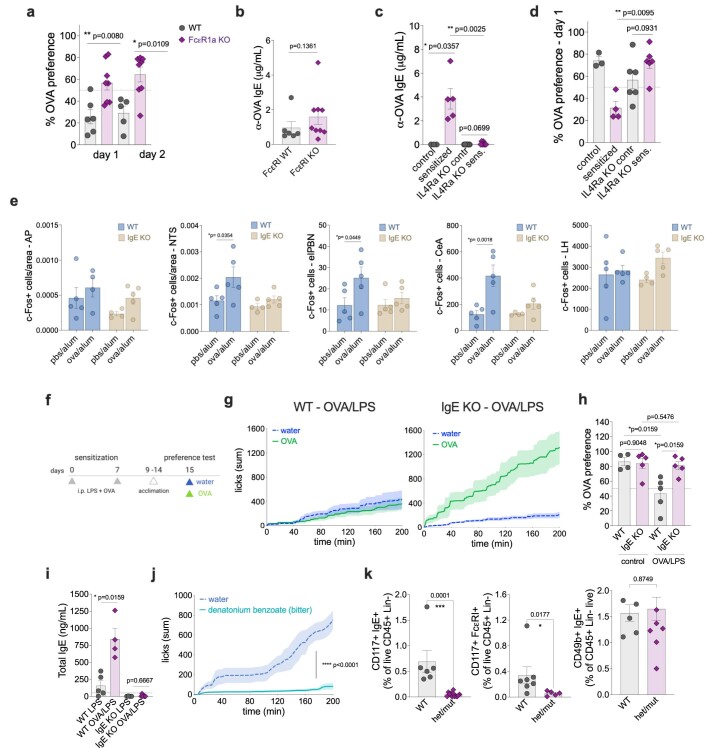
Role of IgE signalling in allergic and non-allergic avoidance. **a**, Drinking preference of BALB/c WT and FcεRI KO to OVA (n = 6 WT and 8 FcεR1 KO mice). **b**, OVA-specific serum IgE in WT and FcεRI KO mice sensitized with OVA/alum (n = 6 WT and 9 FceR1 KO mice). **c**, Serum levels of OVA-specific IgE (n = 3 WT control, 5 WT allergic, 7 IL-4Ra KO control and allergic). **d**, Two-bottle preference test in control and OVA/alum sensitized BALB/c IL-4Ra KO (n = 3 WT control, 4 WT allergic, 6 IL-4Ra KO control and allergic). **e**, Number of cFos+ neurons in the AP, NTS, elPBN, CeA, and LH of control or OVA/alum sensitized WT and IgE KO analysed 90 min after the first oral challenge (n = 4-5 WT or IgE KO control or allergic per group). **f**, Experimental protocol for i.p. sensitization to OVA and lipopolysaccharide (LPS) in WT and IgE KO C57BL/6 mice. **g**, Cumulative licks from C57BL/6 WT (left) or IgE KO (right) mice sensitized i.p. with OVA/LPS (n = 6 WT and 6 IgE KO). **h**, Preference to OVA solution from control (LPS only) and OVA/LPS-sensitized mice (n = 4 WT control, 5 WT allergic, 5 IgE KO control, 5 IgE KO allergic). **i**, Total serum IgE in WT and IgE KO mice sensitized with OVA/LPS (n = 4 WT control, 5 WT allergic, 5 IgE KO control, 5 IgE KO allergic). **j**, Cumulative licks during the two-bottle preference test with water and a solution containing the bitter compound denatonium benzoate in C57BL/6 IgE KO mice sensitized with OVA/alum (*n* = 5 IgE KO). **k**, Frequency of peritoneal (left), intestinal mast cells (centre), and blood basophils (right) by flow cytometry on d16 from control WT or het/mut RMB mice after 3 doses of diphtheria toxin (DT) on days 0, 2, and 4 (n = 6-7 WT and 5-12 RMB het/mut per group). Error bars indicate SEM. *p ≤ 0.05, **p ≤ 0.01, ***p ≤ 0.001. **a-d** and **f-k**, Two-tailed Mann-Whitney test, **e**, Two-way ANOVA with Bonferroni multiple comparisons test. Each panel is representative of at least two independent experiments. **f**, Created with BioRender.com. Source Data

Increased antigen-specific IgE is a hallmark of allergic sensitization and widely used for clinical diagnosis of hypersensitivities^23^. As total and OVA-specific IgE antibodies are increased in the circulation 2 weeks after the first allergen sensitization (Fig. 2a) and C57BL/6 mice, which do not induce strong IgE responses (Extended Data Fig. 3c), also do not promote robust avoidance, we reasoned that IgE might affect behaviour following allergen sensing. Using a genetic approach, we found that sensitization to OVA in IgE-deficient mice did not promote avoidance of OVA solution compared with sensitized littermate wild-type mice (Fig. 2b). Instead, sensitized IgE-knockout (IgE-KO) mice showed increased preference to OVA as compared with their IgE-KO control, unsensitized mice (Fig. 2c). Consistently, sensitized mice deficient in the high-affinity receptor for IgE, FCER1, showed increased preference to OVA compared with wild-type mice (Extended Data Fig. 4a). Serum IgE levels in sensitized FCER1-KO mice were comparable to that of wild-type mice (Extended Data Fig. 4b). By using chimaeric mice, we found that avoidance of OVA was dependent on haematopoietic cells expressing FCER1 (Fig. 2d,e), excluding the possibility of direct allergen sensing by sensory neurons. The lack of OVA avoidance in allergic sensitized mice reconstituted with FCER1-KO haematopoietic cells reflects downstream effects of IgE signalling, as specific IgE levels were equally induced compared with those of WT-reconstituted chimaeric mice (Fig. 2f).

As interleukin-4 (IL-4) is required for IgE production (Extended Data Fig. 4c) and the development of type 2 immune responses^24^, we next tested the role of IL-4 signalling in the induction of avoidance behaviour to OVA. We found that OVA plus alum-sensitized IL-4RA-KO mice did not decrease their consumption of OVA (Extended Data Fig. 4d), suggesting a role for either IL-4 or IL-13, as this cytokine also binds to the same subunit of the IL-4 receptor, in the development of food avoidance. Analysis of FOS staining in the central nervous system indicated that the activation of the NTS, elPBN and CeA induced by the ingestion of allergen is also dependent on IgE (Extended Data Fig. 4e). Notably, IgE-dependent avoidance is not limited to allergic sensitization, as mice sensitized with OVA and lipopolysaccharide—an established non-allergic inflammatory stimulus—also show decreased preference to OVA, which required IgE (Extended Data Fig. 4f–h). OVA plus lipopolysaccharide-sensitized wild-type mice had low but increased levels of IgE (Extended Data Fig. 4i), previously reported in an airway allergic inflammation model^25^. This small increase in IgE levels seems to be sufficient to mediate avoidance behaviour to a conditioned antigen. Finally, OVA plus alum-sensitized IgE-deficient mice showed intact avoidance to a bitter compound, denatonium benzoate (Extended Data Fig. 4j), demonstrating that the lack of OVA avoidance in sensitized wild-type mice is not a generalized phenomenon towards food.

**Extended Data Fig. 5:**
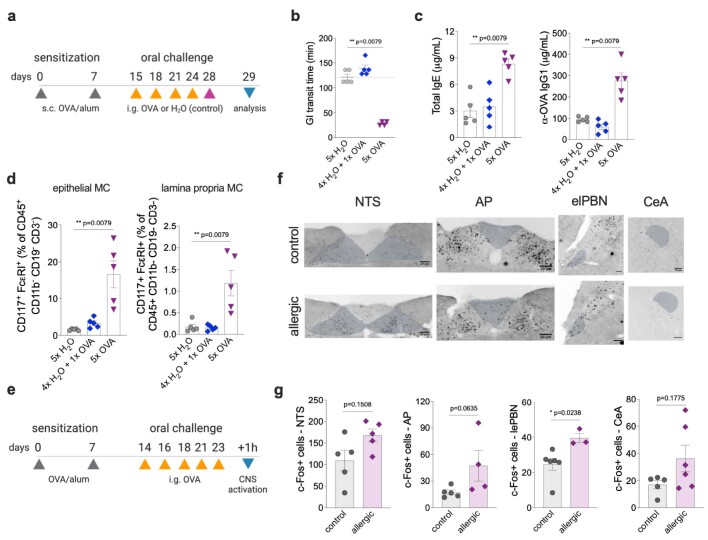
Enteric inflammation and brain activation upon acute or chronic allergen exposure. **a**, Experimental protocol for the development of gut allergic inflammation. BALB/c WT mice were orally challenged with OVA five times (5x OVA group), once on d28 (4x H2O + 1x OVA group) or they received only i.g. water (5x H2O group). Prior to OVA challenges, all mice were s.c. sensitized with OVA and alum. **b**, Gastrointestinal transit time was determined on day 28 upon the fifth oral gavage (n = 4-5 per group). **c**, Total serum IgE (left) and OVA-specific IgG1 (right) antibodies (n = 5 per group). **d**, Flow cytometry analysis of small intestinal mast cells from the epithelial layer (left) and lamina propria (right) (n = 5 per group). **e**, Experimental protocol for brain activation analysis in allergic and control WT BALB/c mice. **f**, Immunofluorescence images of the nucleus tractus solitarius (NTS), area postrema (AP), central amygdala (CeA), and lateral external parabrachial nucleus (elPBN) from control (n = 5-6) or OVA/alum sensitized allergic (n = 3-6) mice using anti-cFos antibody, 90 min after the last OVA challenge. Scale bars = 20 µm. **g**, Number of cFos+ neurons in the NTS, AP, elPBN, and CeA of control or OVA/alum sensitized mice. Graphs show mean ± s.e.m. *p ≤ 0.05, **p ≤ 0.01. Two-tailed Mann-Whitney test. Each panel is representative of at least two independent experiments. **a**,**e**, Created with BioRender.com. Source Data

**Extended Data Fig. 6:**
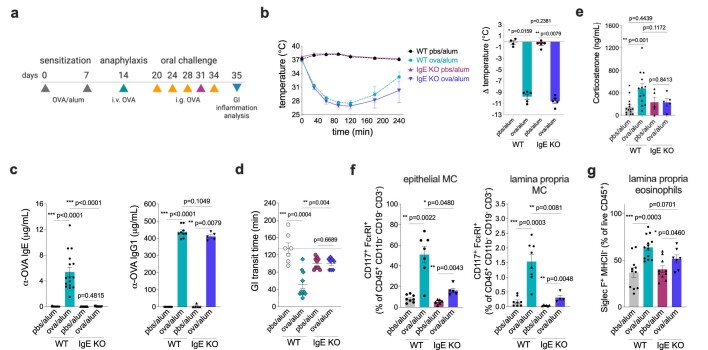
IgE-dependent and independent roles in gut allergic inflammation. **a**, Experimental protocol for the development of gut allergic inflammation and systemic anaphylaxis. Allergic BALB/c WT and littermate IgE KO mice were sensitized with OVA/alum whereas control mice received alum alone. All mice were orally challenged with OVA. **b**, Rectal temperature over time (left) and maximum temperature variation (right) after systemic OVA challenge on d1 (n = 4 WT control and n = 5 allergic, IgE KO control and allergic). **c**, OVA-specific IgE (left) and IgG1 (right) serum antibodies after five oral challenges with OVA (n = 13-17 per group L, n = 5-9 per group R). **d**, Gastrointestinal transit time was determined on day 31 following OVA challenge (n = 7 WT controls, 10 WT allergic, 10 IgE KO controls, 8 IgE KO allergic). **e**, Systemic levels of corticosterone 1h after the fifth OVA challenge in control and OVA/alum sensitized mice (n = 14 WT controls and allergic, 5 IgE KO controls and allergic). **f-g**, Flow cytometry analysis of isolated small intestinal cells from the epithelial layer (left) and lamina propria (centre and right) (n = 8 WT control and allergic, 6 IgE KO control, 5 IgE KO allergic, **f**) (n = 11 WT control, 13 WT allergic, 10 IgE KO control, 7 IgE KO allergic, **g**). **h**, Flow cytometry analysis of enteric mast cells (CD45+ CD19- CD3- CD11b- CD117int FcεRI+ cells) from the small intestine of OVA/alum sensitized BALB/c WT mice after OVA challenges. Controls were sensitized with alum alone and challenged with oral OVA. Graphs show mean ± s.e.m. *p ≤ 0.05, **p ≤ 0.01, ***p ≤ 0.001. Two-tailed Mann-Whitney test. Each panel is representative of at least two independent experiments. **a**, Created with BioRender.com. Source Data

Our findings suggest that IgE is required for the development of avoidance behaviour. However, these antibodies are known to be detrimental and drive allergic symptoms following exposure to food allergens^26^. We suggest that, at early stages, IgE promotes allergic defences such as avoidance behaviour, but following chronic exposure to allergens, IgE leads to disease. To better define the role of IgE on gut allergic inflammation, we first established an experimental model of food allergy. Sensitized mice that received five oral challenges with OVA induced faster gut motility, increased serum IgE and IgG1, enteric mast cell accumulation and CNS activation (Extended Data Fig. 5). Then, systemic anaphylaxis was determined on day 14 in wild-type and IgE-KO mice (Extended Data Fig. 6a). We found that IgE antibodies are not required for the induction of systemic anaphylaxis (Extended Data Fig. 6b), in agreement with a previous report^27^. We confirmed that OVA-specific IgE was induced by wild-type but not by IgE-KO mice; thus, anaphylaxis in IgE-KO mice is probably due to OVA-specific IgG1 antibodies that are increased by allergic sensitization and does not require IgE (Extended Data Fig. 6c). As previously shown^22^, we found that IgE is essential to promote gut motility (Extended Data Fig. 6d) and diarrhoea (not shown) in response to acute allergen exposure in the gastrointestinal tract. IgE is also required for the systemic corticosterone release in allergic mice (Extended Data Fig. 6e). In the intestinal tissue, the accumulation of mast cells in the epithelial and lamina propria compartments, as well as lamina propria eosinophils, was partially dependent on IgE in sensitized and orally challenged mice (Extended Data Fig. 6f,g). These data demonstrate that IgE enhances pathological processes following chronic stimulation with allergen, promoting increased gastrointestinal peristalsis and inflammatory cellular infiltrates in the allergic small intestines.

Mast cells are major gut-resident immune cells implicated in allergies, anaphylaxis, inflammatory bowel disease and abdominal pain^22,23,28,29^. To address their role in the development of avoidance behaviour to allergens, we sensitized RMB mice with OVA plus alum. We depleted mast cells in these mice with three injections of diphtheria toxin before the preference test (Fig. 2g). We confirmed efficient depletion of peritoneal and intestinal mast cells, but not of blood basophils (Extended Data Fig. 4k). Despite being sensitized with OVA plus alum, RMB mice with depleted mast cells showed higher consumption of OVA solution (Fig. 2h) and OVA preference (Fig. 2i) compared with control mice. The systemic levels of IgE antibodies were equally increased in mast cell-depleted mice compared with sensitized wild-type mice (Fig. 2j), suggesting that mast cells are necessary to induce avoidance through IgE sensing of allergens.

**Extended Data Fig. 7:**
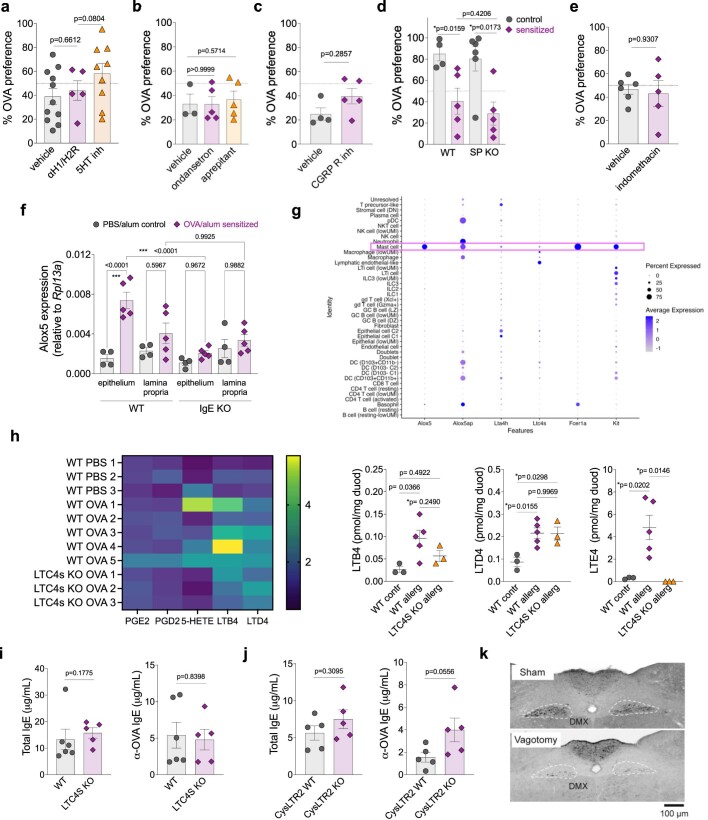
Allergen-induced avoidance behaviour is independent of hallmark mediators but correlated with cysteinyl leukotrienes. **a-c, e**, Drinking preference to OVA solution by OVA/alum sensitized WT BALB/c mice administered with H1 and H2 histamine receptors antagonists (loratadine and famotidine), serotonin synthesis inhibitor (parachlorophenylalanine), serotonin receptor 3 antagonist (ondansetron), substance P receptor NK1 antagonist (aprepitant), CGRP receptor inhibitor (BIBN4096), cyclooxygenase inhibitor (indomethacin), or their respective vehicle solutions prior to preference test (see *Materials and methods* for n for each group*)*. **d**, Preference to OVA in C57BL/6 WT or substance P KO mice orally sensitized with OVA and cholera toxin (n = 4 WT control, 5 WT allergic, 6 Substance P KO control, 5 Substance P KO allergic). **f**, *Alox5* expression levels in the epithelial and lamina propria compartments of the small intestine from BALB/c WT or IgE KO mice sensitized with PBS/alum (control) or OVA/alum (sensitized) (n = 4 WT control, 5 WT allergic, 4 IgE KO control, 5 IgE KO allergic). **g**, Comparison of gene expression in BALB/c intestinal immune cells from published scRNA-seq data of allergic mice (Xu et al., *Immunity*, 2019). **h**, Fold change in duodenal eicosanoids of allergic WT or LTC4S KO mice relative to average of control WT mice (left) and picomoles of detected leukotrienes per mg of duodenal tissue (right) detected by mass spectrometry (n = 3 WT control, 5 WT allergic, 3 LTC4s KO allergic). **i**, Total OVA- specific IgE concentrations in allergic WT or LTC4S KO mice at time of behavioural testing (n = 6 WT allergic, 5 LTC4s KO allergic). **j**, Total and OVA-specific IgE in allergic WT or CysLTR2 KO mice at time of behavioural testing (n = 5 WT allergic, 5 CysLTR2 KO allergic). **k**, Staining of the dorsal motor nucleus of the vagus after i.p. fluorogold injection in sham and vagotomised BALB/c mice to confirm efficiency of subdiaphragmatic vagotomy. Graphs show mean ± s.e.m. *p ≤ 0.05, **p ≤ 0.01, ***p ≤ 0.001. **a-e** and **i-j**, Two-tailed Mann-Whitney test, **f-h**, One-way ANOVA with Turkey’s multiple comparisons test. Each panel is representative of at least two independent experiments. Source Data

Diverse mast cell-derived mediators have been implicated in neuronal excitation in the gastrointestinal tract during intestinal anaphylaxis^11^. Given the rapid change of behaviour observed, we reasoned that the responsible mediators must be either preformed or synthesized de novo after IgE-dependent crosslinking^30^. Histamine and serotonin, released following mast cell degranulation, are two preformed mediators with known roles in mediating itch, pain, diarrhoea and visceral malaise^22,31–33^. We tested the role of the histamine receptors H1 and H2 using the inhibitors loratadine and famotidine. Acute pretreatment with both drugs did not affect OVA preference in control or allergic sensitized mice (Extended Data Fig. 7a), suggesting that histamine might not contribute to food allergen avoidance. Similarly, blockade of serotonin synthesis through 5 days of pretreatment with *para*-chlorophenylalanine led to only a mild and variable effect on avoidance (Extended Data Fig. 7a). We found no effect of pretreatment with the serotonin receptor 5-HT3 antagonist ondansetron as compared to vehicle-treated controls (Extended Data Fig. 7b). Mast cell–nociceptor circuits are well described in the skin, lung and gastrointestinal tract and are proposed to contribute to inflammation and pain perception. Two mediators well known for these interactions are substance P and CGRP^34,35^, which we tested to determine their possible role in mediating avoidance. Using pharmacological (substance P receptor inhibitor, aprepitant) and genetic approaches (substance P-KO mice), we found that substance P did not affect avoidance behaviour to OVA after allergic sensitization (Extended Data Fig. 7b,d). We also found that sensitized mice treated with a CGRP receptor inhibitor (BIBN4096) developed avoidance behaviour towards OVA, comparable to findings for vehicle-treated mice (Extended Data Fig. 7c). So far, our results indicate that histamine, serotonin, substance P and CGRP may not be required for avoidance to food allergens, although this would be important to validate using genetic knockout approaches as the dose and timing of drugs used may have been suboptimal.

**Fig. 3:**
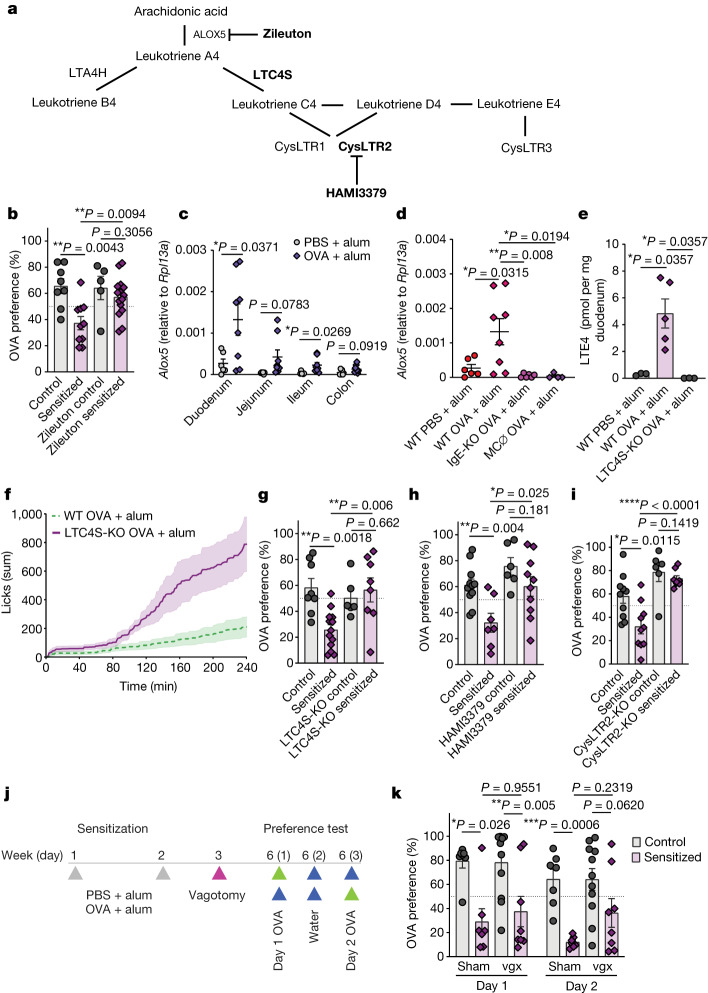
Allergen-induced avoidance behaviour requires cysteinyl leukotrienes. **a**, Leukotriene biosynthetic pathway with targets of inhibitors and knockout mice in bold. **b**, Preference to OVA in OVA + alum-sensitized BALB/c mice. Zileuton was used as an ALOX5 inhibitor 1 h before the preference test (*n* = 8 vehicle control, 10 vehicle allergic, 5 zileuton control, 15 zileuton allergic). **c**, *Alox5* expression across the gastrointestinal tract of control and OVA + alum-sensitized BALB/c mice after allergen oral challenges (*n* = 6 control and 8 allergic). **d**, *Alox5* expression in the duodenum of OVA-sensitized WT, IgE-deficient or mast cell-depleted mice (*n* = 6 WT control, 8 WT allergic, 6 IgE-KO allergic, 4 MC-depleted allergic). **e**, Concentration of LTE4 per milligram of duodenal tissue determined by mass spectrometry in allergic WT and LTC4S-KO mice after oral challenges (*n* = 3 WT control, 5 WT allergic, 3 LTC4S-KO allergic). **f**,**g**, Cumulative licks of OVA (**f**; *n* = 9 WT allergic, 5 LTC4S-KO allergic) and OVA preference (**g**; *n* = 8 WT control, 13 WT allergic, 6 LTC4S-KO control, 8 LTC4S-KO allergic) of sensitized LTC4S-KO or BALB/c control mice. **h**, Preference to OVA in OVA + alum-sensitized mice. HAMI3379 was used as a CysLTR2 inhibitor 1 h before the preference test (first day of preference testing shown; *n* = 11 vehicle control, 7 vehicle allergic, 6 HAMI3379 control, 10 HAMI3379 allergic). **i**, OVA preference of sensitized CysLTR2-KO or BALB/c control mice during the first day of testing (*n* = 10 WT control and allergic, 6 CysLTR2-KO control, 8 CysLTR2-KO allergic). **j**, Schematic protocol for subdiaphragmatic vagotomy in OVA + alum-sensitized BALB/c mice. **k**, OVA preference was determined 3 weeks post vagotomy (vgx; *n* = 7 sham control, 7 sham allergic, 11 vagotomy control, 8 vagotomy allergic). Graphs show mean ± s.e.m. *P ≤ 0.05, **P ≤ 0.01, ***P ≤ 0.001, ****P ≤ 0.0001. **b**,**e**,**g**–**i**,**k**, Two-tailed Man–Whitney test. **c**,**d**, One-way analysis of variance (ANOVA) with Tukey’s multiple-comparison test. Each panel is representative of at least two independent experiments. **j**, Created with BioRender.com. Source Data

In addition to preformed substances, mast cells produce arachidonic acid-derived lipid mediators within minutes after IgE-mediated degranulation^36,37^. Prostanoids and leukotrienes are known to be produced by mast cells and have profound effects on behaviour through actions on nociceptors and vagal neurons^38–41^. These mediators are generated by a series of enzymatic steps controlled by rate-limiting cyclooxygenase and lipoxygenase enzymes (Fig. 3a), respectively. Although cyclooxygenase 1 and 2 inhibition with indomethacin had no effect on the magnitude of avoidance behaviour (Extended Data Fig. 7e), 5-lipoxygenase (ALOX5) inhibition by pretreatment with zileuton significantly increased the preference for OVA in sensitized mice, but did not impact OVA preference in controls (Fig. 3b). Using organ homogenates from wild-type BALB/c mice sensitized and challenged with intragastric OVA, we determined that *Alox5* expression was transcriptionally induced proximally to distally across the intestines, with the highest induction found in the duodenum (Fig. 3c), and in the epithelium (Extended Data Fig. 7f). This pattern of expression was correlated with the geography of intestinal mast cell expansion (Extended Data Figs. 5f and 9b), and detection of *Alox5* transcripts was largely lost in the duodenum of sensitized IgE-KO and mast cell-depleted mice (Fig. 3d and Extended Data Fig. 7f). Analysis of a previously published single-cell RNA-sequencing dataset of intestinal immune cells in sensitized and challenged BALB/c mice revealed that mast cells and basophils, but not other immune cells, expressed all of the transcriptional machinery necessary for leukotriene synthesis (Extended Data Fig. 7g).

ALOX5 oxidizes arachidonic acid to generate leukotriene A4, which is rapidly converted to LTB4 by the enzyme LTA4 hydrolase or conjugated to glutathione to generate LTC4, the parent of all cysteinyl leukotrienes, by LTC4 synthase (LTC4S) (Fig. 3a). To quantify cysteinyl leukotrienes in allergic intestines, we carried out targeted mass spectrometry on duodenal tissue samples of control and allergic wild-type and LTC4S-KO mice. This revealed a 7–25-fold increase in duodenal LTE4 concentrations in allergic mice as compared with controls that was entirely dependent on LTC4S, indicative of intestinal cysteinyl leukotriene production in response to allergen ingestion (Fig. 3e and Extended Data Fig. 7h) whereas PGE2 and PGD2 concentrations were not significantly altered. Mast cells express LTC4S, and sensitized LTC4S-KO mice exhibited increased cumulative licks of and preference for OVA compared with wild-type littermate controls on both days of preference testing, whereas non-sensitized mice were unaffected (Fig. 3f,g). The impact of LTC4S on behaviour is probably downstream of IgE and mast cells, as LTC4S-KO mice induced comparable levels of both total and OVA-specific IgE  as compared to wild-type mice (Extended Data Fig. 7i). Cysteinyl leukotrienes act on three distinct receptors that differ in their affinity for LTC4, LTD4 and LTE4, and their cellular distribution and function^42^. By pharmacological inhibition of CysLTR1 and CysLTR2 using montelukast and HAMI3379 respectively, we found that HAMI3379 largely recapitulated the effect of LTC4S deficiency on the first day of OVA preference testing (Fig. 3h). This effect was confirmed by an increase in OVA preference in sensitized CysLTR2-KO mice relative to wild-type controls, despite similar production of IgE compared with sensitized wild-type mice (Fig. 3i and Extended Data Fig. 7j). The effect of montelukast treatment on allergen avoidance was unable to be conclusively tested owing to an effect of montelukast on OVA intake in non-sensitized mice (data not shown). These data suggest that cysteinyl leukotrienes, probably produced by gastrointestinal mast cells, are required for avoidance behaviour to food allergens. CysLTR2 mediates the acute effect of allergen ingestion on avoidance; however, its effect is not required following re-exposure, suggesting that either CysLTR1 or CysLTR3 signalling and downstream pathways may contribute as well.

Leukotrienes are known to mediate unfavourable sensations through actions on dorsal root ganglion nociceptors and potentially gut-innervating vagal neurons^32,38,39,43^. Following this logic, we probed a role for gut-innervating vagal neurons by conducting subdiaphragmatic vagotomy (Fig. 3j). Successful subdiaphragmatic vagotomy was confirmed by injecting Fluoro-Gold intraperitoneally and finding the absence of dye in the dorsal motor nucleus of the vagus (Extended Data Fig. 7k). However, vagotomy had no significant effect on either the preference to OVA in control mice or the development of avoidance in sensitized mice (Fig. 3k). These findings suggest that vagal afferents individually may not be required for, although they may still contribute to, allergen-induced avoidance behaviour, and instead mast cell-derived signals such as leukotrienes might be sensed through other pathways. Redundant pathways through vagal or nociceptive dorsal root ganglion neurons expressing CysLTR2 may be able to compensate to drive avoidance in each pathway’s individual absence, and it will be crucial to clarify this possibility in the future. Further, subdiaphragmatic vagotomy severs both afferent and efferent motor fibres, and it is possible that this may confound finding a role for such a pathway in the behaviour studied.

**Fig. 4:**
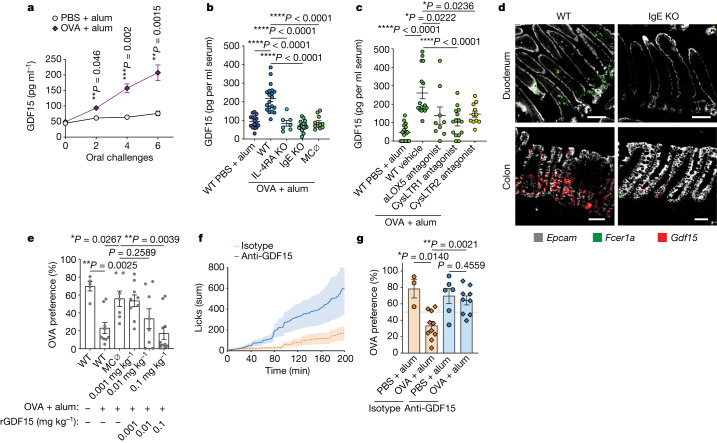
Allergen-induced avoidance requires GDF15. **a**, Serum levels of GDF15 in sensitized BALB/c mice after oral allergen challenges (*n* = 6 control and 10 allergic). **b**, Serum GDF15 in BALB/c WT, IL-4RA KO, IgE-KO and mast cell-depleted (MC ∅) RMB mice after 6 oral challenges with OVA (*n* = 16 WT control, 20 WT allergic, 15 IgE-KO allergic, 11 MC-depleted allergic, 7 IL-4RA-KO allergic). **c**, Serum GDF15 after 6 oral challenges in BALB/c WT mice pretreated with zileuton (ALOX5 antagonist), montelukast (CYSLTR1 antagonist), HAMI3379 (CYSLTR2 antagonist) or vehicle before each challenge (*n* = 15 vehicle control, 16 vehicle allergic, 11 HAMI3379 allergic, 15 montelukast allergic, 9 zileuton allergic). **d**, Expression of *Gdf15* mRNA by RNAscope in the duodenum and colon of allergic (sensitization and five oral challenges) WT or littermate IgE-KO mice (representative of 2 independent experiments with *n* > 3 biological replicates in each group, see Extended Data Fig. 9). Scale bars, 100 µm (for duodenum) and 50 µm (for colon). **e**, OVA preference 1 h after administration of recombinant GDF15 (rGDF15) in mast cell-depleted (MC ∅) RMB mice (*n* = 5 WT control, 9 WT allergic, 7 MC depleted, 9 MC depleted + 0.001 mg kg^−1^, 8 MC depleted + 0.01 mg kg^−1^, 11 MC depleted + 0.1 mg kg^−1^ rGDF15). **f**, Cumulative licks on OVA bottle during two-bottle preference test in sensitized WT mice 5 h after injection with blocking GDF15 antibody or isotype control (*n* = 6 allergic isotype and 6 allergic anti-GDF15). **g**, Sensitized WT mice were injected with blocking GDF15 antibody, and the OVA preference was quantified 5 h later (*n* = 3 isotype control, 10 isotype allergic, 6 anti-GDF15 control, 9 anti-GDF15 allergic). Graphs show mean ± s.e.m. *P ≤ 0.05, **P ≤ 0.01, ****P ≤ 0.0001. **a**–**c**,**e**, One-way ANOVA with Sidak’s multiple-comparison test. **g**, Two-tailed Mann–Whitney *U*-test. Each panel is representative of at least two independent experiments. Source Data

**Extended Data Fig. 8:**
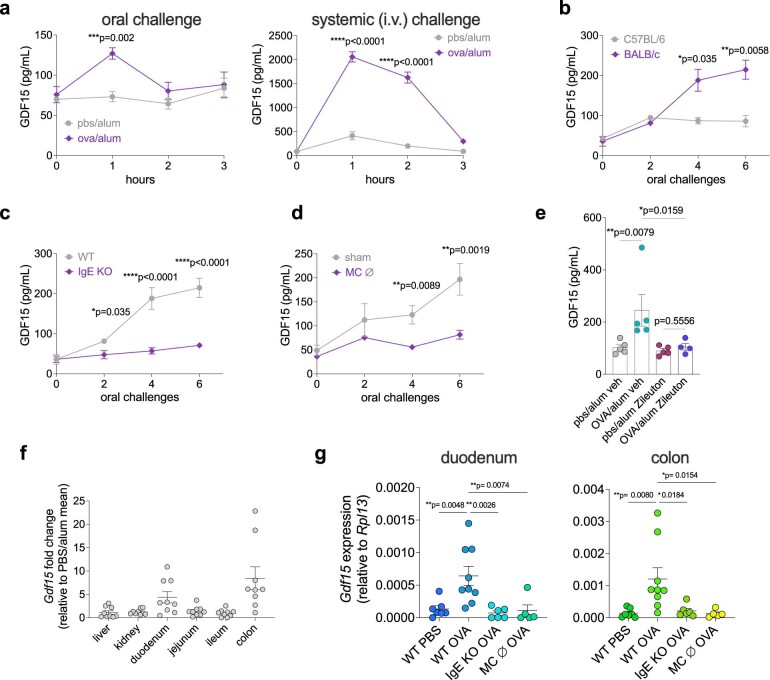
GDF15 is induced upon allergen exposure. **a**, GDF15 serum levels induced by oral (left) (n = 3-8 control and 3-10 allergic per timepoint) or intravenous (right)(n = 4 control and 4 allergic per timepoint) OVA administration in sensitized BALB/c mice. **b-d**, Serum GDF15 induction after oral allergen challenges in OVA/alum sensitized BALB/c and C57BL/6 (n = 4 C57BL/6J, 9 BALB/cJ)(**b**), IgE KO (n = 9 WT, 15 IgE KO) (**c**), or mast cell-depleted RMB (n = 5-8 WT, 5-10 MC Dep mice per timepoint) (**d**) mice. **e**, Induction of serum GDF15 in control and sensitized mice after the administration of the 5- lipoxygenase inhibitor zileuton during oral challenges with OVA (n = 5 control vehicle, 5 allergic vehicle, 5 control zileuton, 4 allergic zileuton). **f**, Fold change in *Gdf15* mRNA transcripts in the intestinal tissues of OVA/alum sensitized BALB/c mice relative to control groups (n = 9 WT allergic normalized to the average transcripts of 7 WT control mice per tissue). **g**, *Gdf15* mRNA transcripts in the duodenum (left) and colon (right) in sensitized mice (n = 7 WT control, 9 WT allergic, 6 IgE KO allergic, 5 MC Dep allergic). Graphs show mean ± s.e.m. *p ≤ 0.05, **p ≤ 0.01, ***p ≤ 0.001. **a-d**, Two-tailed Student’s T-test, **e**, Two-tailed Mann-Whitney test, **g**, One-way ANOVA with Dunett’s multiple comparisons test. Each panel is representative of at least two independent experiments. Source Data

We next examined whether a humoral pathway is involved in allergen avoidance. The area postrema is a sensory circumventricular organ with renowned roles in mediating nausea in the context of noxious stimuli^44^. Growth and differentiation factor 15 (GDF15) is a transforming growth factor-β superfamily cytokine produced during conditions of inflammation and cell stress that acts on the area postrema and NTS by binding to its receptor, GFRAL, to mediate conditioned flavour avoidance and anorexia^45^. Serum GDF15 levels were, in fact, induced following food allergen challenge (Fig. 4a and Extended Data Fig. 8a, left), and this induction was amplified with the increasing number of challenges (Fig. 4a and Extended Data Fig. 8a, left). Serum GDF15 levels were even more robustly induced following systemic OVA challenge (Extended Data Fig. 8a, right). The induction of GDF15 was found to be dependent on background strain, with BALB/c, but not C57BL/6, mice exhibiting elevated serum levels in response to oral allergen challenge (Extended Data Fig. 8b). This induction in BALB/c was entirely dependent on IL-4RA, IgE- and FCER1A-expressing cells and could be partially prevented by pretreating mice with zileuton (ALOX5 inhibitor), montelukast (CysLTR1 inhibitor) or HAMI3379 (CysLTR2 inhibitor) before each challenge (Fig. 4b,c and Extended Data Fig. 8c–e), suggesting that mast cell production of cysteinyl leukotrienes may be involved in GDF15 secretion.

**Extended Data Fig. 9:**
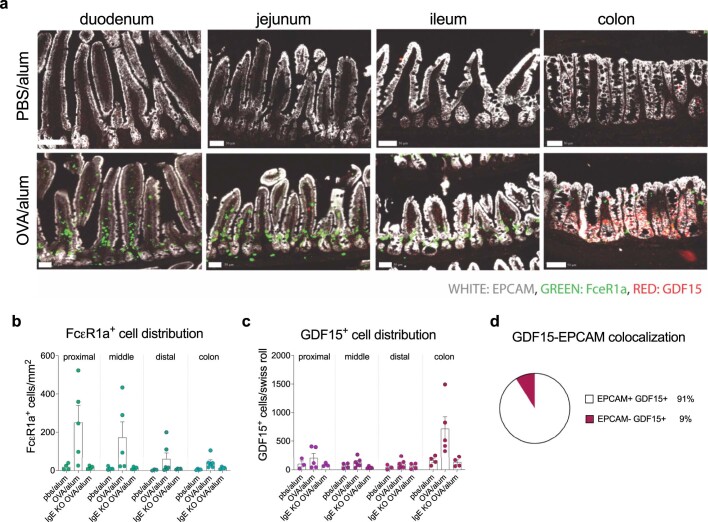
GDF15 originates from colonic epithelial cells. **a**, Fcεr1a (green), EPCAM (grey), and GDF15 (red) transcripts across intestinal tissues in OVA/alum BALB/c sensitized and control mice by RNAScope. Scale bars =  50 µm. **b-c**, Analysis of intestinal distribution of FcεR1 expressing cells (**b**) and GDF15 expressing cells (**c**) from control and allergic sensitized WT or IgE KO mice (n = 4 WT control, 5 WT allergic, 4 IgE KO allergic per group). Quantification was performed after RNAscope technique. **d**, Colocalization analysis of GDF15 expressing colonic cells and cells expressing the epithelial cell marker, EPCAM utilizing sum of all WT 5 allergic mice. Graphs show mean ± s.e.m. *p ≤ 0.05, **p ≤ 0.01, ***p ≤ 0.001. Two-way ANOVA by Genotype/Sensitization Status and Anatomical Region. Representative of two independent experiments. Source Data

Using organ homogenates from sensitized mice challenged intragastrically with OVA, we found that *Gdf15* was induced on the transcriptional level mainly in the duodenum and colon (Extended Data Fig. 8f). *Gdf15* induction in these tissues was dependent on IgE antibodies and mast cells (Extended Data Fig. 8g). Using RNAscope in situ hybridization with probes specific for *Gdf15*, *Fcer1a* and *Epcam*, we demonstrated that *Gdf15* was induced by the colon after intragastric OVA challenges, with a lower magnitude of induction in the small intestine (Fig. 4d and Extended Data Fig. 9a–c). This was unexpected, as mast cell expansion occurred in a proximal-to-distal pattern across the small and large intestine. In fact, *Gdf15* transcripts showed little (<1%) overlap with *Fcer1a* transcripts, and instead colocalized primarily (93%) with EPCAM^+^ cells (Extended Data Fig. 9d). These GDF15^+^ epithelial cells were found to form close contact with mast cells and were primarily found in the colonic crypt andtransit-amplifying zone. Notably, similar colonic induction patterns were previously described following treatment of mice with metformin^46^, and p53 induction by DNA damage, which rapidly dividing cells are more susceptible to, is capable of inducing anorexia through a GDF15-dependent mechanism^47^. Hence, food allergen ingestion induces colonic epithelial *Gdf15* transcription through IgE, mast cells and leukotrienes.

**Extended Data Fig. 10:**
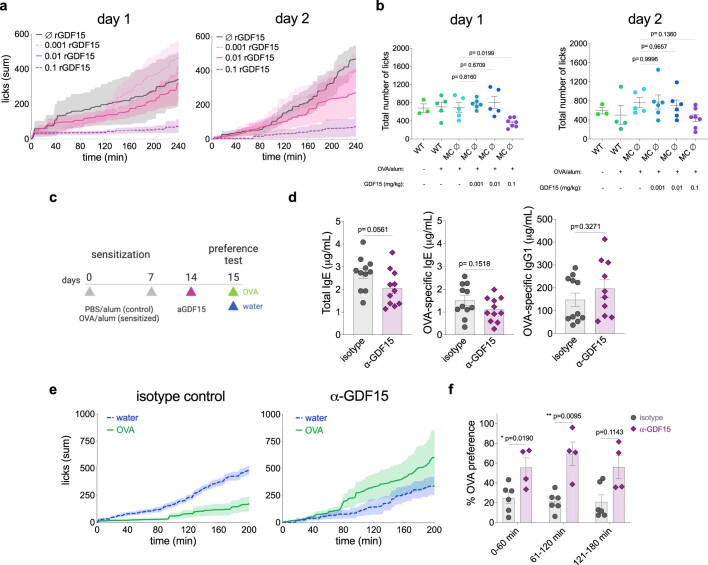
GDF15 blockade reduces allergen avoidance. **a**, Cumulative licks of OVA over time and (**b**) total licks of OVA and water in mast cell-depleted RMB mice retreated with rGDF15 (mg/kg) from one experiment pertaining to Fig. 4e (n = 3 WT control, 5 WT allergic, 4 MC Dep, 6 MC Dep + 0.001mg/kg, 6 MC Dep + 0.01 mg/kg, 7 MC Dep + 0.1 mg/kg rGDF15). **c**, BALB/c mice were sensitized with OVA/alum and treated with a mouse GDF15 blocking antibody or isotype control 5 h prior to two bottle preference testing. **d**, Total IgE (left), OVA-specific IgE (centre), and OVA-specific IgG1 (right) antibodies after neutralizing GDF15 antibody treatment (n = 11 isotype, 10 aGDF15). **e**, Cumulative licks during the two-bottle preference test of isotype control (left) or GDF15 blocking antibody-treated (right) mice (n = 6 isotype, 6 aGDF15). **f**, OVA preference of OVA/alum sensitized mice that received either GDF15 blocking antibody or isotype control 5 h prior to the behavioural assay (n = 6 isotype, 4 aGDF15). Graphs show mean ± s.e.m. *p ≤ 0.05, **p ≤ 0.01, ***p ≤ 0.001. **a-b**, One-way ANOVA with Dunnett’s multiple comparisons test, **d**, Two-tailed Student’s t-test, **f**, Two-tailed Mann-Whitney test. Each panel is representative of at least two independent experiments. **c**, Created with BioRender.com. Source Data

To determine whether GDF15 was able to impact OVA avoidance in our behavioural paradigm, and whether mast cells were necessary for these effects, we sensitized BALB/c wild-type and littermate control RMB mice and depleted mast cells with diphtheria toxin as described (Fig. 2g). Mast cell-depleted mice were then injected with increasing doses of recombinant GDF15 immediately before each day of OVA preference testing. We found that GDF15 rescued allergen avoidance in mast cell-depleted mice, consistent with GDF15 being downstream of mast cell activation (Fig. 4e). This avoidance was dose dependent, leading to a partial response at 0.01 mg kg^−1^ and one similar to sensitized BALB/c mice at 0.1 mg kg^−1^ (Extended Data Fig. 10a). The doses necessary to drive OVA avoidance were higher than that induced by intragastric OVA challenge (Fig. 4a,b), and on the first, but not second, day of recombinant GDF15 treatment, this was associated with a significant reduction in total licks in this group (Extended Data Fig. 10b). Thus, we sought to directly test whether GDF15 was necessary for promoting avoidance of OVA using anti-GDF15 neutralizing antibody. We sensitized wild-type mice with OVA plus alum and treated them 5 h before each day of OVA preference testing with isotype control or anti-GDF15 (Extended Data Fig. 10c). Both isotype- and anti-GDF15-treated allergic mice failed to avoid OVA on day 1 of preference testing, suggesting a potential effect of antibody administration per se on the OVA avoidance (data not shown). On day 2 of the preference test, however, blocking GDF15 led to increased consumption of OVA in allergic sensitized mice relative to isotype control treatment (Fig. 4f,g). The increase in OVA preference could not be explained by differences in antibody titres, as the levels of total and OVA-specific IgE as well as OVA-specific IgG1 were similarly induced in isotype- and anti-GDF15-treated groups (Extended Data Fig. 10d). Neutralization of GDF15 increased preference to OVA in sensitized mice within 60 min and lasted throughout the 3 h of the second trial (Extended Data Fig. 10e,f). GDF15 seems to be necessary for allergen avoidance, yet it is incapable of driving avoidance alone at concentrations comparable to the allergen challenge, suggesting that other signals may synergize with GDF15 to promote avoidance. Given our findings, cysteinyl leukotrienes are potential mediators able to explain this effect. Their actions on multiple pathways, including potentially CysLTR2-expressing vagal and nonpeptidergic nociceptors in addition to the induction of epithelial GDF15, may explain the profound lack of avoidance seen following LTC4S deficiency, which was only partially recapitulated by deficiency of CysLTR2. Thus, here we describe an unexpected mast cell–epithelial circuit that generates allergen avoidance through a cysteinyl leukotriene- and GDF15-dependent mechanism.

Together, these findings demonstrate that immune sensing of allergens leads to the generation of avoidance behaviour. We suggest that avoidance behaviour is a defence strategy aimed at minimizing harmful effects of exposure to noxious substances, including allergens. Our findings suggest that detection of allergens by IgE antibodies expands sensory capacity of the nervous system and provides a mechanism to evaluate the quality of the food and other environmental factors. This conclusion adds to a growing body of evidence indicating that the immune detection of noxious stimuli is an important source of sensory information that drives corresponding behaviours^48^. It also adds to the growing evidence of bidirectional functional interactions between the immune and nervous systems^49,50^.

## Methods

### Animals

All animal care and experimentation were approved by the Institutional Animal Care and Use Committee of Yale University School of Medicine and consistent with the National Institutes of Health, USA, guidelines. Mouse lines were interbred in our facilities to obtain the final strains described in the text. Genotyping was carried out according to the protocols established for the respective strains by The Jackson Laboratories or published by the donating investigators. Mice were maintained at the Yale University animal facilities in temperature- (22 °C) and humidity-controlled rooms, in a 12-h light/dark cycle with free access to standard chow diet (Teklad 2018S, Envigo) and water.

Female mice at 6–10 weeks of age were used for all experiments. BALB/cJ (000651), C57BL/6J (000664), C57BL/6 FCER1-KO (B6.129S2(Cg)-*Fcer1a*^*tm1Knt*^/J, 010512), BALB/c Il4ra-KO (BALB/c-*Il4ra*^*tm1Sz*^/J, 003514)^51^, C57BL/6 substance P-KO (B6.Cg-*Tac1*^*tm1Bbm*^/J, 004103)^34^, LTC4S-KO (C.129S7(B6)-*Ltc4s*^*tm1Blam*^/J, 0309539)^52^ and CysLTR2-KO (C.B6-*Cysltr2*^*tm1Ykn*^/J, 031718)^53^ mice were purchased from The Jackson Laboratories and maintained in our facilities. BALB/c IgE-KO mice were provided by H. C. Oettgen (Harvard University), RMB (B6. Ms4a2^tm1Mal^) mice^54^ were provided by P. Launay (Université Paris Diderot) and C57BL/6 *Trpm5*^*−/−*^ mice were provided by W. Garret (Harvard University). RMB, FCER1-KO, IgE-KO or *Trpm5*^*−/−*^ mice were backcrossed more than eight times onto BALB/cJ or C57BL/6J for this study. We used littermate controls in all experiments.

Chimaeric mice were generated following a standard protocol^55^. C57BL/6J wild-type or FCER1-KO mice were used as donors in bone marrow transplant experiments. C57BL/6J wild-type recipient mice underwent a lethal total-body irradiation with two doses of 500 rad (Gammacell 40 ^137^Cs γ-irradiation source), with an interval of 3 h between the first and the second irradiation. Fresh, unseparated bone marrow cells (10 × 10^6^ per mouse) were injected into the tail vein of the irradiated recipient mice 4 h after lethal irradiation. Chimaerism efficiency was checked by flow cytometry 8 weeks post-irradiation and transplant using peripheral blood, and reconstituted mice were used 2 months after bone marrow transplantation.

For mast cell depletion, sensitized RMB mice were injected intraperitoneally (i.p.) with 0.05 mg kg^−1^ of diphtheria toxin (Sigma-Aldrich D0564) three times every other day as previously described^54^, starting on day 28 after the first allergic sensitization. As this protocol was efficient at depleting mast cells even in heterozygous RMB mice, we used both heterozygous and homozygous mutants as mast cell-depleted models.

For drug trials, famotidine (Sigma-Aldrich F6889)^56^ and loratadine (Sigma-Aldrich, L9664)^57^ were both i.p. injected to a final concentration of 10 mg kg^−1^ for 2 days before the preference test. *p*-chlorophenylalanine (Tocris, 0938) was injected i.p. for 5 consecutive days before the preference test to a final concentration of 100 mg kg^−1^ (ref. ^58^). Zileuton (Tocris, 3308)^39^ was used at 50 mg kg^−1^ in 0.6% methylcellulose by gavage, 1 h before each day of preference testing. HAMI3379 (Cayman 10580)^59^ or montelukast (Cayman 35779)^60^ was used at 0.4 and 10 mg kg^−1^ respectively in sterile PBS intraperitoneally, 1 h before each day of preference testing. Ondansetron hydrochloride (Tocris, 2891)^61^ was injected i.p. at 1 mg kg^−1^ 1 h before preference test. Aprepitant (Sigma-Aldrich SML2215)^62^ was i.p. injected 1 h before preference test at 5 mg kg^−1^. Indomethacin (Sigma-Aldrich, I7378) was orally administered in the drinking water for 5 consecutive days before the preference test at a final concentration of 0.02 mg ml^−1^. The CGRP-selective antagonist, BIBN4096 (Tocris, 4561)^63^, was i.p. injected at 30 mg kg^−1^ 50 min before preference test. All drugs were solubilized following the manufacturer’s instructions. All control groups received the appropriate vehicle solutions.

Sample size for drug trials pertaining to Extended Data Fig. 7a–c,e were as follows—Extended Data Fig. 7a: 11 vehicle allergic, 5 aH1R and aH2R allergic, 9 *p*-chlorophenylalanine allergic; Extended Data Fig. 7b: 3 vehicle allergic, 5 ondansetron allergic, 5 aprepitant allergic; Extended Data Fig. 7c: 4 vehicle allergic, 5 BIBN4096 allergic; Extended Data Fig. 7e: 6 vehicle allergic, 5 indomethacin allergic.

For antibody treatment experiments, animals were injected intravenously through the retro-orbital sinus with control antibody to keyhole limpet haemocyanin (clone LTF-2) or GDF15-blocking antibody (patent ID: WO2014100689A1, gift from Dr. Hui Tian)^64^ both at 10 mg kg^−1^ in 0.1 ml PBS 6 h before preference test. Purified mouse GDF15 (patent ID: WO2012138919A2) was injected i.p. 1 h before the preference test at concentrations ranging from 0.001 to 0.1 mg kg^−1^.

### Allergic sensitization and challenges

Animals were sensitized subcutaneously on days 0 and 7 with 0.25 mg kg^−1^ endotoxin-free OVA (BioVendor 321001) adsorbed in 50 mg kg^−1^ alum gel (Invivogen vac-alu-250) and diluted to a final volume of 0.2 ml in PBS pH 7.4. Controls received all of the above except for OVA (referred to as PBS + alum).

For oral sensitization experiments, mice were given oral gavages on days 0 and 7 with 5 mg of grade III OVA with 0.5 mg kg^−1^ of cholera toxin (List Biologicals 100B, lot nos. 10165A1 and 10165A2) in a final volume of 0.25 ml of 0.2 M sodium bicarbonate buffer. Both sensitization and challenge were carried out around zeitgeber time 4 (ZT4).

For allergen challenges, all groups received 5 oral gavages with 40 mg grade III OVA (Sigma-Aldrich A5378) in 0.25 ml of normal drinking water on days 14, 18, 21, 24 and 28, unless otherwise stated. For Figs. 3 and 4 and Extended Data Figs. 5 and 6, all groups received 5 oral gavages with 50 mg grade III OVA (as above) in 0.2 ml PBS on days 14, 16, 18, 20 and 22.

### Preference test

Drinking behaviour was determined using the two-bottle preference test in custom-built lickometer cages, on the basis of the commonly used sucrose preference test^65^ and following previous studies^13,17^. Two days after the last sensitization, on day 9, mice were individually placed into the lickometer cages and transferred to the temperature-controlled test room. During the 5 days of adaptation, mice were provided continuous exposure to two normal water bottles. Baseline measurements started on the last day of adaptation, day 14. On day 15, each mouse was given one bottle of water and one bottle containing 1% OVA (grade II, Sigma A5253), unless otherwise stated, 30 min before the lights turned off. The number of licks in each bottle was recorded periodically (1 or 5 min, as indicated) overnight. On day 16 at ZT2, OVA bottles were switched back to normal water bottles and the baseline preference was measured until the next day. On day 17, mice were provided with two new bottles 30 min before the lights turned off: one containing water and the other containing a fresh solution of 1% OVA. The bottles’ positions were switched to account for potential place preference. Preference results are expressed as cumulative licks over time or as percentage OVA preference, calculated using the area under the curve of cumulative licks from the OVA bottle divided by the total cumulative licks (OVA + water). Total solution intake was also measured by calculating the difference in solution weight before and after the preference test. Mice had ad libitum access to chow diet.

Avoidance specificity was tested using bovine serum albumin (Fisher BP1600-1). We used denatonium benzoate (Sigma D5765) as the bitter compound for positive control of avoidance behaviour.

### Brain tissue preparation and immunohistochemistry

Brains were collected 90 min after the first or fifth oral challenge with OVA. To minimize gavage-induced stress, we administered sham gavages with normal drinking water for 3 days before the final allergen challenge. Mice were deeply anaesthetized with isoflurane (Covetrus) and were transcardially perfused with PBS followed by freshly prepared 4% paraformaldehyde (PFA) in PBS. Dissected brains were kept in 4% PFA at 4 °C for 48 h, washed 3 times in PBS and transferred to a 30% sucrose in PBS solution for 2 days and then sliced into 40-μm-thick coronal sections (area postrema, dorsal motor nucleus of the vagus, NTS and PBN) and 100-μm-thick sections (lateral hypothalamus and CeA) using a Leica CM3050 S cryostat (Leica Biosystems). Briefly, the sections were permeabilized with PBS with 0.3% Triton X-100 for 30 min at room temperature, and then blocked in PBS with 0.3% Triton X-100 and 10% normal donkey serum in 0.3 M glycine for 1 h at room temperature. Blocking was followed by incubation with rabbit monoclonal anti-FOS primary antibody (1:1,000 dilution, Cell Signalling 2250S) or rabbit polyclonal anti-Fluoro-Gold primary antibody (1:1000 Fluorochrome) in the same blocking solution overnight for 16 h and then with Alexa Fluor 594-conjugated donkey anti-rabbit IgG secondary fluorescent antibody (1:500 dilution, Invitrogen A21207) for 2 h at room temperature. After being washed with the permeabilization solution again, the sections were mounted on slides and visualized by using a fluorescent All-In-One Keyence microscope (model BZ-X710, Keyence). Images were taken using the ×4 objective. Brains regions were defined on the basis of the Allen Mouse Brain Atlas reference atlas (https://mouse.brain-map.org/) and processed using the open-source Fiji-ImageJ software^66^. FOS^+^ cells were manually quantified by a blinded investigator throughout the entire procedure. In the experiment using wild-type and IgE-KO mice, FOS immunolabelling was carried out using a whole-brain clearing protocol^67,68^ (1:1,000, FOS, rabbit polyclonal antibody, 226 003, Synaptic Systems). Images were acquired on the light sheet microscope (LaVision Ultramicroscope II). For FOS quantification and analysis, the ClearMap2 pipeline was used^68^, with manual (and blinded) validation of the cell counts in the NTS, area postrema and PBN. Parameters for FOS detection were adjusted before the FOS analysis. All images were processed in the Imaging Core Facility, at Yale University.

### Antibody quantification

Serum levels of total IgE and OVA-specific IgE antibodies were determined by sandwich enzyme-linked immunosorbent assay (ELISA). For total IgE, ELISA-grade plates (490012-252, VWR) were coated overnight at 4 °C with 2 mg ml^−1^ of anti-mouse IgE (553413, clone R35-72, BD Pharmingen) in 0.1 M sodium carbonate buffer pH 9.5. Plates were blocked with 1% bovine serum albumin (Fisher BP1600-1) for 2 h at room temperature. Serum from sensitized and control mice was diluted up to 1:100 and incubated for 2 h at room temperature. Purified mouse IgE (BD Biosciences 557079 for BALB/c and 557080 for C57BL/6) was used as standard at the highest concentration of 10 ng ml^−1^ followed by twofold dilutions to create a standard curve. Afterwards, 500 ng ml^−1^ of biotin-conjugated anti-IgE detection antibodies (553419, clone R35-118, BD Biosciences) was incubated for 1 h at room temperature followed by another incubation with diluted HRP-conjugated streptavidin (554066, BD Biosciences) for 30 min at room temperature. Then plates were incubated in the dark at room temperature with TMB substrate reagent (555214, BD Biosciences) and the colour was checked every 3 min. Plates were read at 450 nm immediately after adding stop solution (3 M H_2_SO_4_). Between each step, plates were washed five to seven times with 0.05% Tween-20 in PBS. Serum concentrations of OVA-specific IgE were assayed by the same ELISA method. Purified mouse anti-OVA IgE (MCA2259, clone 2C6, Bio-Rad) was used as standard with the highest concentration at 100 ng ml^−1^. Capture antibodies were the same as for total IgE assay and 8 mg ml^−1^ of biotinylated OVA (OVA1-BN-1, Nanocs) was used for detection. OVA-specific IgG1 antibodies in the serum were assayed by direct ELISA. Plates were coated overnight at 4 °C with 20 mg ml−1 of grade V OVA (A5503, Sigma-Aldrich) in coating buffer (0.1 M sodium carbonate pH 9.5). After the blocking step, serum samples were diluted up to 1:10,000 and incubated for 1 h at room temperature. Purified mouse BALB/c IgG1 (557273, clone MOPC-31C, BD Biosciences) was used as standard with the highest concentration at 100 ng ml^−1^. Biotin-conjugated mouse IgG1 (553441, clone A85-1, BD Biosciences) was used for detection at 100 ng ml^−1^. The HRP-conjugated streptavidin, substrate reagents, blocking and washing solutions used for IgG1 ELISA were the same as described above for IgE ELISAs.

### Oral and systemic anaphylaxis

To determine the occurrence of active systemic anaphylaxis, sensitized mice were challenged intravenously through the retro-orbital sinus with 5 mg kg^−1^ of grade V OVA (A5503, Sigma-Aldrich) 14 days after the first sensitization at ZT4. For oral anaphylaxis, mice were administered with 40 mg of grade III OVA (A5378, Sigma-Aldrich) intragastrically. Rectal temperature was measured every 30 min for 4 h after challenge using a probe (Thermalert TH-5).

### Complete subdiaphragmatic vagotomy

Mice were sensitized with OVA and alum on days 0 and 7 as described above. Mice were placed on a liquid diet 4 to 5 days before to promote survival and recovery. Mice were i.p. treated with bupivacaine (2 mg kg^−1^), buprenorphine (1 mg kg^−1^) and meloxicam (5 mg kg^−1^). Mice were anaesthetized with 4% isoflurane for induction and then transferred under the microscope and maintained at 1–1.5% isoflurane during the surgery. An abdominal midline incision was made through the skin and the intraperitoneal wall. The stomach was exteriorized to expose the oesophagus. Two blunted and bent 19G needles were gently placed at the proximal and distal end of the oesophagus to isolate it from the remaining tissue. The vagus nerves could be observed running dorsal and ventral to the oesophagus. The nerves were incised at the most proximal end of the oesophagus using curved fine forceps. Control sham-operated mice received the same surgical procedure except for the incision of the nerves. The blunted needles were removed, and the intraperitoneal cavity and the skin layer were sutured. Post-surgery animals were given a liquid diet for ten days with moistened chow also provided five days post-surgery. Animals were then placed on a normal chow diet and used for experimental purposes two weeks post-surgery. Histological verification of vagotomy was confirmed using an i.p. injection of 0.1% Fluoro-Gold (Fluorochrome), followed by examination of its presence in the dorsal motor nucleus of the vagus 2 weeks after injection.

### Gastrointestinal motility

Gastrointestinal transit time was assessed immediately following the fourth oral challenge with intragastric OVA, on day 24 after allergic sensitization at ZT3. Mice were gavaged with a 0.25 ml solution with 6% carmine red (C1022, Sigma-Aldrich), 0.5% methylcellulose (M0512, Sigma-Aldrich) and 40 mg grade III OVA. After oral gavage and for the duration of the assay, mice were individually placed in clean cages containing normal bedding. Mice had free access to food and water and were monitored for the occurrence of diarrhoea. The gastrointestinal transit time was measured as the time between oral gavage and the appearance of the first faecal pellet containing the red carmine dye. Mice were grouped together at the end of the assay.

### Epithelial cell and lamina propria isolation

Single-cell suspensions of small intestinal epithelium and lamina propria were prepared as described previously^69,70^. Briefly, the small intestine was isolated and opened longitudinally. Its contents were then rinsed in PBS following removal of Peyer’s patches. The tissue was then cut into 2–3-cm segments and incubated in RPMI medium (ThermoFisher) containing 5 mM EDTA, 0.145 mg ml^−1^ dithiothreitol and 3% FBS at 37 °C with 5% CO_2_ for 20 min with agitation. Pieces of intestine were washed in RPMI containing 2 mM EDTA to separate the epithelial fraction. This fraction was then subjected to a 30% Percoll density gradient by centrifugation (GE17-0891-01, Sigma-Aldrich). Lamina propria digestion was carried out using 0.1 mg ml^−1^ Liberase TL (Roche no. 540102001) and 0.5 mg ml^−1^ DNAse (DN25, Sigma-Aldrich) in RPMI for 30 min at 37 °C with 5% CO_2_. Digested tissue was sequentially strained through 70 mM and 40 mM strainers, and then washed in RPMI containing 3% FBS, and cells were then stained for further analysis.

### Peritoneal lavage and isolation of blood leukocytes

Mice were euthanized by CO_2_ inhalation. Blood was collected by cardiac puncture, and then subjected to three rounds of red blood cell lysis using ACK lysis buffer (Lonza no. BP10-548E), and the single-cell suspension was stained for flow cytometry. Mice were also injected intraperitoneally with 4 ml of complete RPMI medium and 1 ml of air. The medium was aspirated from the peritoneal cavity using a glass Pasteur pipette. The collected fluid was centrifuged, and the single-cell suspension was stained for flow cytometry.

### Flow cytometry

Single-cell suspensions were treated with anti-CD16/32 (Fc block; 14-9161-73, ThermoFisher) and stained with the live/dead marker ethidium monoazide bromide (ThermoFisher no. E1374) in 2% FBS in PBS or the Zombie Yellow Fixable Viability Kit (Biolegend no. 423103) in PBS. The following antibodies were used at a concentration of 1 mg ml^−1^ except where otherwise indicated: APC–Cy7–CD117 (clone 2B8; Biolegend no. 105826), APC/eFluor780–MHCII (clone M5/114.15.2; eBioscience 47-5321-82), APC/eFluor780–CD19 (clone eBio1D3; eBioscience 47-0193-82), APC/eFluor780–CD4 (clone RM4-5; Invitrogen 47-0042-82), PE–FCER1 (clone MAR-1; eBioscience 12-5898-82), PE–SiglecF (clone E50-2440; BD Pharmingen no. 552126), PE–Gata3 (clone TWAJ; eBioscience no. 12-9966-42), eFluor450-CD45 (clone 30-F11; eBioscience no. 48-0451-82), eFluor450–FCER1 (clone MAR-1; eBioscience no. 48-5898-82), APC–CD11b (clone M1/70; eBioscience no. 17-0112-82), APC–Ly6c (clone HK1.4; eBioscience no. 17-5932-82), APC–TCRb (clone H57-597; Biolegend no. 109212), APC–SA (eBioscience no. 17-4317-82), APC–MHCII (clone M5/114.15.2; eBioscience no. 17-5321-82), Alexa700-CD3 (clone 17A2; Biolegend no. 100216), Alexa700–CD45 (clone 30-F11; Biolegend no. 103128), Alexa700–CD19 (clone 6D5; Biolegend no. B189284), PE/Cy–CD3e (clone 145-SC11, eBioscience no. 25-0031-82), PE/Cy7–Ly6G (clone RB6-8C5; eBioscience no. 25-5931-82), PE/Cy7–CD117 (clone 2B8; eBioscience no. 25-1171-82), PE/Cy7–Tbet (clone eBio4B10; eBioscience no. 25-5825-82), PE/Cy7–CD4 (clone GK1.5; Biolegend no. 100422), PE/Cy7–CD11b (clone M1/70; eBioscience no. 25-0112-82), PE/Cy7–CD45R (clone RA3-6B2; eBioscience no. 25-0452-82), PE/Cy7–NK1.1 (clone PK136; BD Pharmingen no. 552878), FITC–CD11c (clone N418; eBioscience no. 11-0114-85), FITC–IgE (clone R35-72; BD Pharmingen no. 553415), FITC–CD11b (clone M1/70; eBioscience no. 11-0112-85), FITC–CD19 (clone eBio1D3; eBioscience no. 11-0193-82), FITC–Gr1 (Ly6G/Ly6c; clone RB6-8C5; Biolegend no. 108406), FITC–NK1.1 (clone PK136; Biolegend no. 108706), FITC–Ter119 (clone Ly76; Biolegend no. 116206), FITC–CD49b (clone DX5; Biolegend no. 108906), FITC–Lin (clones 145-2C11, RB6-8C5, RA3-6B2, Ter119, M1/70; Biolegend no. 133301), FITC–MHCII (clone M5/114.15.2; eBioscience no. 11-5321-82), biotin–IgE (clone R35-72; BD Pharmingen no. 553414), BV711–F4/80 (clone T45-2342; BD Horizon no. 565612), BV421–CD11b (clone M1/70; BD Horizon no. 562605), BV421–RORgt (clone Q31-378; BD Horizon no. 562894), BUV395–CD45 (clone 30-F11; BD Horizon no. 564279), BUV737–CD90.2 (clone 53-2.1; BD Bioscience no. 741701) and PECy5.5–Foxp3 (clone FJK-16s; eBioscience no. 35-5773-82). Cells were fixed with 1.6% PFA (Electron Microscopy Sciences, no. 15710). Flow cytometry was carried out using a BD LSRII analyser equipped with the following lasers: 355 nm (ultraviolet), 405 nm (violet), 488 nm (blue) and 633 nm (red). Data were analysed using FlowJo software v10.8.1. Gates were drawn according to fluorescence minus one control. Representative gating for all experiments can be found in Supplementary Fig. 1. Supplementary Fig. 1a–f denotes small intestinal immune cell populations, Supplementary Fig. 1g demonstrates gating of peritoneal mast cells, and Supplementary Fig. 1h shows gating strategy for identification of blood basophils.

### RNAscope

For RNAscope experiments, fluorescence in situ hybridization was carried out on FFPE tissues as previously described^71^. Briefly, mice were sensitized as above and challenged intragastrically 6 times every other day with 50 mg of grade III OVA. One hour after the sixth gavage, mice were euthanized by CO_2_ asphyxiation and the small intestine and colon were collected. Tissues were flushed with PBS, followed by 4% PFA, opened longitudinally and fixed overnight at room temperature. The following day, the small intestine was divided into thirds, and all tissues were rolled using the Swiss roll technique and subsequently embedded in paraffin. RNAscope was carried out using the RNAscope Multiplex Flourescent v2 kit (ACD 323110) using probes specific for GDF15 (ACD 318521), EPCAM (ACD 418151-C2) and FCER1A (ACD 511331-C3) following the manufacturer’s instructions for FFPE tissues with fluorescent detection through Opal 620, Opal 520 and Opal 570, respectively (Akoya Biosciences FP1495001KT, FP1487001KT, SKU FP1488001KT). Slides were counterstained with 4′,6-diamidino-2-phenylindole (DAPI). Quality of tissue RNA was confirmed, and background threshold was established using positive and negative control probes (ACD 320881, ACD320871). Images were acquired on a Leica STP 6000 fluorescence microscope using tile-scans of entire Swiss roll sections at ×20 resolution. FCER1A, EPCAM and GDF15 colocalization analysis was carried out in Qupath (QuPath Quantitative Pathology & Bioimage Analysis, v3.0) on the basis of the fluorescent threshold of cells detected by DAPI positivity.

### Eicosanoid quantification by mass spectrometry

Proximal duodenal tissue samples (3 cm immediately distal to the pylorus) from control or sensitized wild-type or littermate control LTC4S-KO mice were collected 1 h after the fifth intragastric OVA challenge. Targeted eicosanoid quantification was carried out at the UCSD Lipidomics Core. Briefly, eicosanoids were purified using strata-x polymeric reverse-phase columns (8B-S100-UBJ Phenomenex), reconstituted in buffer A, and analysed by reversed-phase ultraperformance liquid chromatography with mass spectrometry using a Sciex 6500 Qtrap mass spectrometer using previously described methods^72^.

### Histological quantification of intestinal mast cells

Mast cells were quantified histologically by CAE staining as previously described^22^. Briefly, the middle 10 cm of small intestine was collected 1 h after the seventh OVA gavage, and flushed once intraluminally with PBS, and then once with 10% NBF. Intestines were then open longitudinally and fixed overnight on Whatman paper in 10% NBF at room temperature and subsequently rolled into Swiss rolls as previously described. The intestines were then dehydrated, embedded in paraffin, cut into 5-μm sections, and stained for naphthol chloroacetate esterase stain by the Yale Pathology Tissue Service. Mast cells were imaged and quantified by manually counting five independent ×10 fields of view for CAE^+^ cells in QuPath and averaging them to obtain an average per independent sample. Each independent biological sample is represented as an individual point on Extended Data Fig. 3h.

### GDF15 quantification

Serum for all GDF15 measurements was taken from mice 1 h after 50 mg intragastric OVA challenge unless otherwise noted. GDF15 levels in the serum were measured by ELISA (R&D, MGD150).

### RNA extraction and quantification

For tissue RNA extraction, tissues were collected into RNA STAT-60 RNA isolation reagent (Amsbio) and disrupted by bead homogenization (Omni, INC). RNA was extracted using the Direct-zol RNA Mini Kit (Zymo, R2051). cDNA synthesis was carried out using MMLV reverse transcriptase (Clontech) with oligo(dT) primers. Quantitative PCR with reverse transcription was carried out on the CFX384 Real-Time System (Bio-Rad) using PerfeCTa SYBR Green Supermix (Quanta Biosciences) and transcripts were normalized to *Rpl13a*.

### Statistics

Statistical analyses were carried out in GraphPad Prism software v9.5.0. Data were analysed with the Mann–Whitney *U*-test (two experimental groups) or one-way ANOVA with multiple-comparison test. Statistical significance is defined as *P < 0.05, **P < 0.01, ***P < 0.001. Nonparametric statistical analyses were used throughout the manuscript and all data are mean ± s.e.m., unless stated otherwise.

### Reporting summary

Further information on research design is available in the Nature Portfolio Reporting Summary linked to this article.

## Online content

Any methods, additional references, Nature Portfolio reporting summaries, source data, extended data, supplementary information, acknowledgements, peer review information; details of author contributions and competing interests; and statements of data and code availability are available at 10.1038/s41586-023-06362-4.

## Supplementary information

Supplementary Fig. 1 Gating strategy for immune cell populations.

Reporting Summary

## Data Availability

All data will be made freely available upon reasonable request. Single-cell RNA-sequencing data for intestinal immune cells during food allergy in BALB/cJ mice (Extended Data Fig. 7g) can be accessed at the National Center for Biotechnology Information Gene Expression Omnibus, accession number GSE124880 (ref. ^73^). Alternatively, processed single-cell RNA-sequencing data for this same study can be found at https://portals.broadinstitute.org/single_cell/study/fasi-immune-mouse-small-intestine. Allen Mouse Brain Atlas reference atlas is available from https://atlas.brain-map.org. ClearMap 2.0 with WobblyStitcher, TubeMap and CellMap is available at https://github/ChristophKirst/ClearMap2 (refs. ^68,74^). Source data are provided with this paper.

## Source data

Source Data Fig. 1
Source Data Fig. 2
Source Data Fig. 3
Source Data Fig. 4
Source Data Extended Data Fig. 1
Source Data Extended Data Fig. 2
Source Data Extended Data Fig. 3
Source Data Extended Data Fig. 4
Source Data Extended Data Fig. 5
Source Data Extended Data Fig. 6
Source Data Extended Data Fig. 7
Source Data Extended Data Fig. 8
Source Data Extended Data Fig. 9
Source Data Extended Data Fig. 10
