# Supplementary material for: Immune sensing of food allergens promotes avoidance behaviour
Source: Nature. 2023 Jul 12;620(7974):643–50. doi: 10.1038/s41586-023-06362-4 (PMC10432274; doi:10.1038/s41586-023-06362-4)
Supplement: Supplementary file 1 — Gating strategy for immune cell populations. [file 41586_2023_6362_MOESM1_ESM.pdf]

nature portfolio

https://doi.org/10.1038/s41586-023-06362-4

Supplementary information

Immune sensing of food allergens promotes avoidance behaviour

In the format provided by the
authors and unedited

Supplementary Information Figure Guide

SI Figure 1. Gating strategy for immune cell populations.

Supplementary Information Figure Legend

Supplementary Fig. 1|Gating strategies for flow cytometric analysis. a, Gating strategy to identify live CD45+ singlet enteric cells presented in Extended Data Fig. 2b, 2c, 4k, and 6f. **b,** Gating strategy to identify enteric mast cells (CD19- CD3- CD11b- CD117+ FcεRI+) presented in Extended Data Fig. 2b, 2c, 4k, and 6f. **c,** Gating strategy to identify enteric T cell subsets (Lineage- CD4+ TCRβ+, Foxp3+ or Gata3+ or Tbet+ or RORgt+) or ILC2s (Lineage- CD4- TCRβ- CD90.2+ Gata3+) presented in Extended Data Fig. 2b and 2c. **d,** Gating strategy to identify enteric dendritic cells (CD11c+ MHCII+), macrophages (F4/80+ MHCII+), or monocytes (F4/80- CD11b+ Ly6c+) presented in Extended Data Fig. 2b and 2c. **e,** Gating strategy to identify enteric neutrophils (MHCII- CD11b+ Gr-1+) presented in Extended Data Fig. 2b and 2c. **f,** Gating strategy to identify enteric eosinophils (SiglecF+ MHCII-) presented in Extended Data Fig. 2b, 2c, and 6g. **g,** Gating strategy to identify peritoneal mast cells (live CD45+ Lineage- CD117+ IgE+ singlets) as presented in Extended Data Fig. 4k. **h,** Gating strategy to identify blood basophils (live CD45+ Lineage- CD49b+ IgE+ singlets) presented in Extended Data Fig. 4k. Each panel is representative of at least two independent experiments.

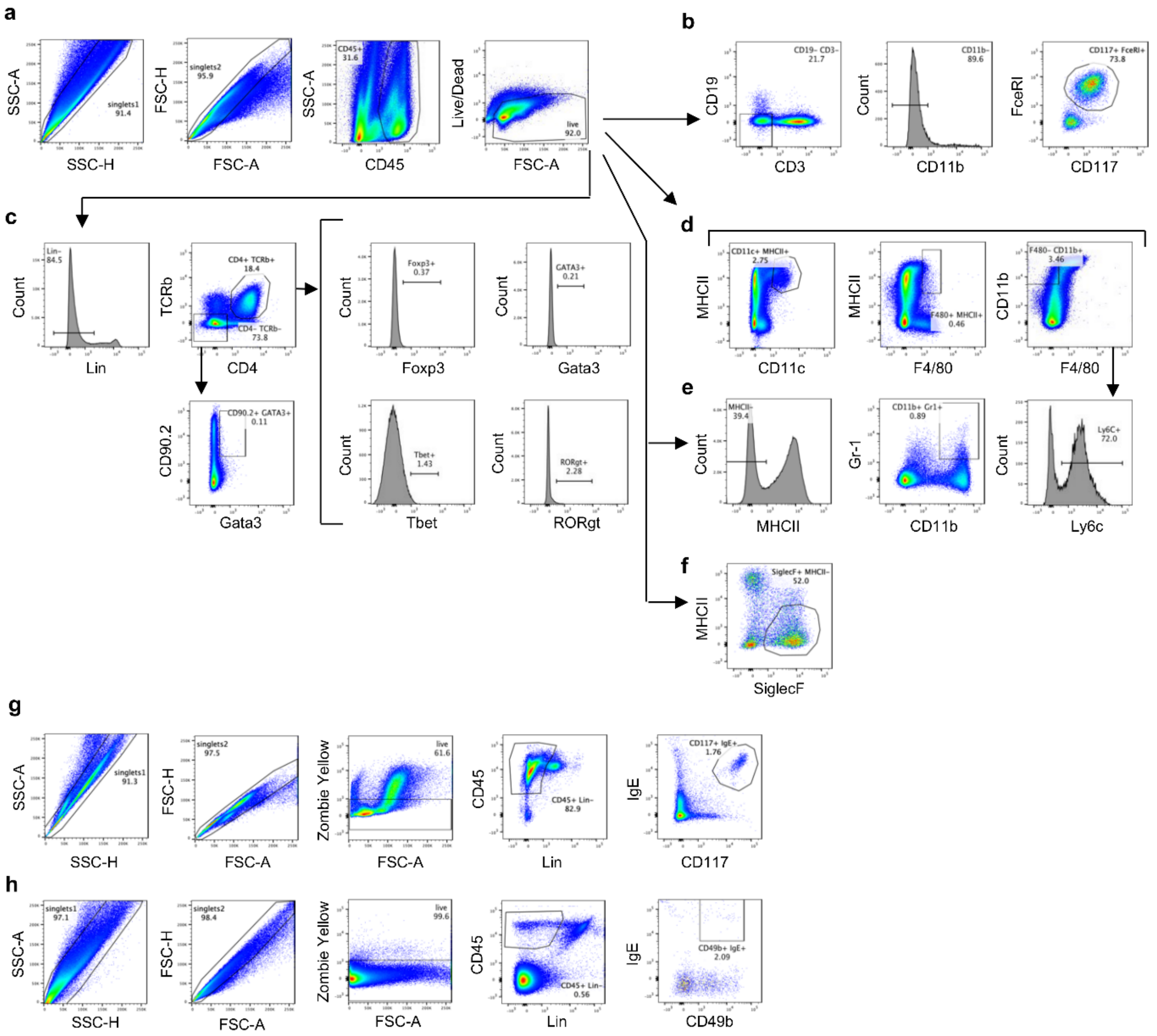

Supplementary Figure 1
