# Supplementary material for: Immune sensing of food allergens promotes avoidance behaviour
Source: Nature. 2023 Jul 12;620(7974):643–50. doi: 10.1038/s41586-023-06362-4 (PMC10432274; doi:10.1038/s41586-023-06362-4)
Supplement: Supplementary file 2 — Reporting Summary [file 41586_2023_6362_MOESM2_ESM.pdf]

nature portfolio

Corresponding author(s): Ruslan Medzhitov

Last updated by author(s): 05/25/2023

Reporting Summary

Nature Portfolio wishes to improve the reproducibility of the work that we publish. This form provides structure for consistency and transparency in reporting. For further information on Nature Portfolio policies, see our Editorial Policies and the Editorial Policy Checklist.

Statistics

For all statistical analyses, confirm that the following items are present in the figure legend, table legend, main text, or Methods section.

n/a	Confirmed	
☐	☒	The exact sample size (*n*) for each experimental group/condition, given as a discrete number and unit of measurement
☐	☒	A statement on whether measurements were taken from distinct samples or whether the same sample was measured repeatedly
☐	☒	The statistical test(s) used AND whether they are one- or two-sided<br>*Only common tests should be described solely by name; describe more complex techniques in the Methods section.*
☒	☐	A description of all covariates tested
☐	☒	A description of any assumptions or corrections, such as tests of normality and adjustment for multiple comparisons
☐	☒	A full description of the statistical parameters including central tendency (e.g. means) or other basic estimates (e.g. regression coefficient) AND variation (e.g. standard deviation) or associated estimates of uncertainty (e.g. confidence intervals)
☐	☒	For null hypothesis testing, the test statistic (e.g. *F*, *t*, *r*) with confidence intervals, effect sizes, degrees of freedom and *P* value noted<br>*Give P values as exact values whenever suitable.*
☒	☐	For Bayesian analysis, information on the choice of priors and Markov chain Monte Carlo settings
☒	☐	For hierarchical and complex designs, identification of the appropriate level for tests and full reporting of outcomes
☒	☐	Estimates of effect sizes (e.g. Cohen's *d*, Pearson's *r*), indicating how they were calculated

Our web collection on statistics for biologists contains articles on many of the points above.

Software and code

Policy information about availability of computer code

Data collection	No software was used.
Data analysis	Fiji-Image J, QuPath v3.0, Flowjo 10.8.1, Graphpad prism v9.5.0

For manuscripts utilizing custom algorithms or software that are central to the research but not yet described in published literature, software must be made available to editors and reviewers. We strongly encourage code deposition in a community repository (e.g. GitHub). See the Nature Portfolio guidelines for submitting code & software for further information.

Data

Policy information about availability of data

All manuscripts must include a data availability statement. This statement should provide the following information, where applicable:

- Accession codes, unique identifiers, or web links for publicly available datasets
- A description of any restrictions on data availability
- For clinical datasets or third party data, please ensure that the statement adheres to our policy

The data availability statement was added to the manuscript.

Human research participants

Policy information about studies involving human research participants and Sex and Gender in Research.

Reporting on sex and gender	N/A
Population characteristics	N/A
Recruitment	N/A
Ethics oversight	N/A

Note that full information on the approval of the study protocol must also be provided in the manuscript.

Field-specific reporting

Please select the one below that is the best fit for your research. If you are not sure, read the appropriate sections before making your selection.

☒ Life sciences ☐ Behavioural & social sciences ☐ Ecological, evolutionary & environmental sciences

For a reference copy of the document with all sections, see nature.com/documents/nr-reporting-summary-flat.pdf

Life sciences study design

All studies must disclose on these points even when the disclosure is negative.

Sample size	No formal sample size calculations were performed prior to experimentation. Sample size was determined based on prior literature performing similar measurements (Mirotti et al.; 2009).
Data exclusions	Exclusion of data was pre-established for the two bottle preference tests. Samples with cumulative licks of 0 or over 100/minute were excluded.
Replication	Each experiment reported in the manuscript was repeated at least two times with independent cohorts of mice with >3 technical replicates of individual mice per cohort.
Randomization	Allocation of individual animals in control or experimental groups was random.
Blinding	Fos-positive cells in the brain were manually quantified by a blinded investigator throughout the entire procedure. For all other assays, blinding was not required as they do not reflect subjective scorings.

Reporting for specific materials, systems and methods

We require information from authors about some types of materials, experimental systems and methods used in many studies. Here, indicate whether each material, system or method listed is relevant to your study. If you are not sure if a list item applies to your research, read the appropriate section before selecting a response.

Materials & experimental systems

n/a	Involved in the study	
☐	☒	Antibodies
☒	☐	Eukaryotic cell lines
☒	☐	Palaeontology and archaeology
☐	☒	Animals and other organisms
☒	☐	Clinical data
☒	☐	Dual use research of concern

Methods

n/a	Involved in the study	
☒	☐	ChIP-seq
☐	☒	Flow cytometry
☒	☐	MRI-based neuroimaging

Antibodies

Antibodies used	APC-Cy7-CD117 (clone 2B8; Biolegend #105826)<br>APC/eFluor780-MHCII (clone M5/114.15.2; eBioscience 47-5321-82)<br>APC/eFluor780-CD19 (clone eBio1D3; eBioscience 47-0193-82)<br>APC/eFluor780-CD4 (clone RM4-5; Invitrogen 47-0042-82)

PE-FceRI (clone MAR-1; eBioscience 12-5898-82)
PE-SiglecF (clone E50-2440; BD Pharmingen #552126)
PE-Gata3 (clone TWAJ; eBioscience #12-9966-42)
eFluor450-CD45 (clone 30-F11; eBioscience #48- 0451-82)
eFluor450-FceRI (clone MAR-1; eBioscience #48-5898-82)
APC-CD11b (clone M1/70; eBioscience #17-0112-82)
APC-Ly6c (clone HK1.4; eBioscience #17-5932-82)
APC-TCRb (clone H57-597; Biolegend #109212)
APC-SA (eBioscience #17-4317-82)
APC-MHCII (clone M5/114.15.2; eBioscience #17-5321-82)
Alexa700-CD3 (clone 17A2; Biolegend 15 #100216)
Alexa700-CD45 (clone 30-F11; Biolegend #103128)
Alexa700-CD19 (clone 6D5; Biolegend #B189284)
PE/Cy7-CD3e (clone 145-SC11, eBioscience #25-0031-82)
PE/Cy7-Ly6G (clone RB6-8C5; eBioscience #25-5931-82)
PE/Cy7-CD117 (clone 2B8; eBioscience #25-1171-82)
PE/Cy7-Tbet (clone eBio4B10; eBioscience #25-5825-82)
PE/Cy7-CD4 (clone GK1.5; Biolegend #100422)
PE/Cy7-CD11b (clone M1/70; eBioscience #25-0112-82)
PE/Cy7-CD45R (clone RA3-6B2; eBioscience #25-0452-82)
PE/Cy7-NK1.1 (clone PK136; BD Pharmingen #552878)
FITC-CD11c (clone N418; eBioscience #11-0114-85)
FITC-IgE (clone R35-72; BD Pharmingen #553415)
FITC-CD11b (clone M1/70; eBioscience #11-0112-85)
FITC-CD19 (clone eBio1D3; eBioscience #11-0193-82)
FITC-Gr1 (Ly6G/Ly6c) (clone RB6-8C5; Biolegend#108406)
FITC-NK1.1 (clone PK136; Biolegend #108706)
FITC-Ter119 (clone Ly76; Biolegend #116206)
FITC-CD49b (clone DX5; Biolegend #108906)
FITC-Lin (clones 145- 2C11, RB6-8C5, RA3-6B2, Ter-119, M1/70; Biolegend #133301)
FITC-MHCII (clone M5/114.15.2; eBioscience #11-5321-82)
biotin-IgE (clone R35-72; BD Pharmingen #553414)
BV711-F4/80 (clone T45-2342; BD Horizon #565612)
BV421-CD11b (clone M1/70; BD Horizon #562605)
BV421-RORgt (clone Q31-378; BD Horizon #562894)
BUV395-CD45(clone 30-F11; BD Horizon #564279)
BUV737-CD90.2 (clone 53-2.1; BD Bioscience #741701)
PECy5.5-Foxp3 (clone FJK-16s; eBioscience #35-5773-82)
Rabbit monoclonal anti-c-Fos primary antibody (Cell Signaling #2250S)
Rabbit Polyclonal anti-fluoro-gold (Fluorochrome)
Alexa Fluor 594-donkey anti-rabbit IgG secondary antibody (Invitrogen, #A-21207)
Purified Rat Anti-Mouse IgE (clone R35-72; BD Biosciences #553413)
Purified Mouse IgE,k Isotype Control (clone C38-2; BD Biosciences #557079)
Purified Mouse IgE,k Isotype Control (clone C48-2; BD Biosciences #557080)
Biotin Rat Anti-Mouse IgE (clone R35-118; BD Biosciences #553419)
Streptavidin-HRP (BD Biosciences #553419)
Ovalbumin antibody (anti-OVA IgE) (clone 2C6; BioRad MCA2259)
Ovalbumin, Biotin Labeled (Nanocs OVA1-BN-1)
Purified Mouse IgG1,k Isotype Control (clone MOPC-31C; BD Biosciences #557273)
Biotin Rat Anti-Mouse IgG1 (clone A85-1; BD Biosciences #553441)

Validation

All antibodies were previously validated for flow cytometry by the manufacturer, unless otherwise noted.
CD16/CD32 (Fc block) was validated by ThermoFisher using THP-1 cells.
Ethidium monoazide bromide was validated by ThermoFisher using Jurkat cells.
Zombie Yellow Fixable Viability Kit was validated by Biolegend using mouse splenocytes.
CD11c-FITC was validated by eBioscience using mouse splenocytes.
IgE-FITC was validated in our lab by using mouse peritoneal cells.
CD11b-FITC was validated by eBioscience using mouse bone marrow cells.
CD19-FITC was validated by eBioscience using mouse splenocytes.
Gr1 (Ly6G/Ly6c)-FITC was validated by Biolegend using mouse bone marrow cells.
NK1.1-FITC was validated by Biolegend using mouse splenocytes.
Ter119-FITC was validated by Biolegend using mouse bone marrow cells.
CD49b-FITC was validated by Biolegend using mouse splenocytes.
Lin-FITC was validated by Biolegend using mouse bone marrow cells.
MHCII-FITC was validated by eBioscience using mouse splenocytes.
SiglecF-PE was validated by BD Pharmingen using mouse bone marrow cells.
FceRI-PE was validated by eBioscience using the mouse mast cell line, MC/9.
Gata3-PE was validated by eBioscience using mouse thymocytes.
Ly6c-APC was validated by eBioscience using mouse splenocytes.
CD11b-APC was validated by eBioscience using mouse bone marrow cells.
TCRb-APC was validated by Biolegend using mouse splenocytes.
MHCII-APC was validated by eBioscience using mouse splenocytes.
SA-APC was validated by eBioscience using flow cytometry.
IgE-biotin was validated in our lab by using mouse peritoneal cells.
CD3e-PE/Cy7 was validated by eBioscience using mouse splenocytes.
Ly6G-PE/Cy7 was validated by eBioscience using mouse bone marrow cells.

CD117-PE/Cy7 was validated by eBioscience using mouse bone marrow cells.
Tbet-PE/Cy7 was validated by eBioscience using normal human peripheral blood cells.
CD4-PE/Cy7 was validated by Biolegend using mouse splenocytes.
CD11b-PE/Cy7 was validated by eBioscience using mouse bone marrow cells.
CD45R-PE/Cy7 was validated by eBioscience using mouse splenocytes.
NK1.1-PE/Cy7 was validated by BD Pharmingen using mouse splenocytes.
MHCII-APC/Cy7 was validated by eBioscience using mouse splenocytes.
CD19-APC/Cy7 was validated by eBioscience using mouse splenocytes.
CD4-APC/Cy7 was validated by eBioscience using mouse splenocytes.
CD117-APC/Cy7 was validated by Biolegend using mouse bone marrow cells.
F4/80-BV711 was validated by BD Horizon using mouse splenocytes.
CD11b-BV421 was validated by BD Horizon using mouse bone marrow cells.
RORgt-BV421 was validated by BD Horizon using mouse thymocytes.
CD45-BUV395 was validated by BD Horizon using mouse splenocytes.
CD3-Alexa700 was validated by Biolegend using mouse splenocytes.
CD19-Alexa700 was validated by Biolegend using mouse splenocytes.
CD45-Alexa700 was validated by Biolegend using mouse splenocytes.
CD45-e450 was validated by eBioscience using mouse bone marrow cells.
FceRI-e450 was validated by eBioscience using a mouse mast cell line, MC/9.
CD90.2-BUV737 was validated in our lab using mouse splenocytes.
Foxp3-PECy5.5 was validated by eBioscience using mouse splenocytes.
c-fos primary and secondary antibodies were validated using mouse brains.
Purified Rat Anti-Mouse IgE was validated by ELISA.
Purified Mouse IgE,� Isotype Control was validated by ELISA.
Purified Mouse IgE,� Isotype Control was validated by ELISA.
Biotin Rat Anti-Mouse IgE was validated by ELISA.
Streptavidin-HRP was validated by ELISA.
Ovalbumin antibody (anti-OVA IgE) was validated by ELISA.
Ovalbumin, Biotin Labeled was validated by ELISA.
Purified Mouse IgG1,� Isotype Control was validated by ELISA.
Biotin Rat Anti-Mouse IgG1 was validated by ELISA.

Animals and other research organisms

Policy information about studies involving animals; ARRIVE guidelines recommended for reporting animal research, and Sex and Gender in Research

Laboratory animals	Female mice at 6-10 weeks of age were used for all experiments. BALB/cJ (000651), C57BL/6J (000664), C57BL/6 FceRI KO (B6.129S2(Cg)-Fcer1atm1Knt/J, 010512), DdblGATA1 (C.129S1(B6)-Gata1tm6Sho/J, 005653), BALB/c Il4ra KO (BALB/c-Il4ratm1Sz/J, 003514), and C57BL/6 substance P KO (B6.Cg-Tac1tm1Bbm/J, 004103) mice were purchased from The Jackson Laboratories and maintained in our facilities. BALB/c IgE KO were generously provided by H. C. Oettgen (Harvard University), RMB (B6. Ms4a2tm1Mal) were generously provided by P. Launay (Université Paris Diderot), and C57BL/6 Trpm5-/- mice were provided by W. Garret (Harvard University). RMB, Fc�RI KO, IgE KO, or Trpm5-/- mice were backcrossed more than eight times onto BALB/cJ or C57BL/6J for this study. We used littermate controls in all experiments.
Wild animals	The study did not involve wild animals.
Reporting on sex	Since testosterone was shown to have a suppressive effect on mast cell activation and secretion of anaphylactic mediators (Mackey et al., 2019), we did not use male mice for this study.
Field-collected samples	The study did not involve samples collected from the field.
Ethics oversight	All animal care and experimentation were approved by the Institutional Animal Care and Use Committee of Yale University School of Medicine and consistent with the National Institutes of Health, USA, guidelines.

Note that full information on the approval of the study protocol must also be provided in the manuscript.

Flow Cytometry

Plots

Confirm that:

☒ The axis labels state the marker and fluorochrome used (e.g. CD4-FITC).

☒ The axis scales are clearly visible. Include numbers along axes only for bottom left plot of group (a 'group' is an analysis of identical markers).

☒ All plots are contour plots with outliers or pseudocolor plots.

☒ A numerical value for number of cells or percentage (with statistics) is provided.

Methodology

Sample preparation	Single-cell suspensions were treated with anti-CD16/32 (Fc block) and stained with one of two live/dead markers: (1)

Sample preparation

ethidium monoazide bromide in 2% FBS in PBS or (2) Zombie Yellow in PBS. Antibodies were used at a concentration of 1 ug/mL. Cells were fixed with 1.6% paraformaldehyde.

Instrument

BD LSRII analyser equipped with the following lasers: 355 nm (UV), 405 nm (violet), 488 nm (blue), and 633 nm (red).

Software

BD FACS Diva software was used to collect data. Data was analyzed using FlowJo software.

Cell population abundance

Cell sorting was not performed.

Gating strategy

Enteric Mast Cell gating: SSC-H vs. SSC-A and FSC-A vs. FSC-H to exclude doublets, CD45+ EMA-CD3- CD19- CD11b- CD117+ FceRI+
Peritoneal Mast Cell gating: SSC-H vs. SSC-A and FSC-A vs. FSC-H to exclude doublets, CD45+ EMA- CD3- CD19- CD11b- CD117+ FceRI+
Dendritic Cell gating: SSC-H vs. SSC-A and FSC-A vs. FSC-H to exclude doublets, CD45+ EMA- CD11c+ MHCII+
Macrophage gating: SSC-H vs. SSC-A and FSC-A vs. FSC-H to exclude doublets, CD45+ EMA- F4/80+ MHCII+
Monocyte gating: SSC-H vs. SSC-A and FSC-A vs. FSC-H to exclude doublets, CD45+ EMA- F4/80- CD11b+ Ly6c+
Neutrophil gating: SSC-H vs. SSC-A and FSC-A vs. FSC-H to exclude doublets, CD45+ EMA- CD11b+ Gr1+ MHCII-
Eosinophil gating: SSC-H vs. SSC-A and FSC-A vs. FSC-H to exclude doublets, CD45+ EMA- SiglecF+ CD11b+ MHCII-
ILC2 gating: SSC-H vs. SSC-A and FSC-A vs. FSC-H to exclude doublets, CD45+ EMA- Lin- CD4- TCRb- CD90.2+ Gata3+
CD4+ T Cell gating: SSC-H vs. SSC-A and FSC-A vs. FSC-H to exclude doublets, CD45+ EMA- Lin- CD4+ TCRb+
RORgt+ T Cell gating: SSC-H vs. SSC-A and FSC-A vs. FSC-H to exclude doublets, CD45+ EMA- Lin- CD4+ TCRb+ RORgt+
Tbet+ T Cell gating: SSC-H vs. SSC-A and FSC-A vs. FSC-H to exclude doublets, CD45+ EMA- Lin- CD4+ TCRb+ Tbet+
Gata3+ T Cell gating: SSC-H vs. SSC-A and FSC-A vs. FSC-H to exclude doublets, CD45+ EMA- Lin- CD4+ TCRb+ Gata3+
Foxp3+ T Cell gating: SSC-H vs. SSC-A and FSC-A vs. FSC-H to exclude doublets, CD45+ EMA- Lin- CD4+ TCRb+ Foxp3+
Basophil gating: SSC-H vs. SSC-A and FSC-A vs. FSC-H to exclude doublets, CD45+ EMA- Lin-CD49b+ IgE+.

☒ Tick this box to confirm that a figure exemplifying the gating strategy is provided in the Supplementary Information.
